# Spiking neurons-based facial emotion recognition: a comparative analysis of leaky and a quadratic integrated neuron for the unprocessed dataset

**DOI:** 10.3389/fncom.2026.1869643

**Published:** 2026-07-17

**Authors:** Anu Roopa Devi Sekar, Ruban Nersisson

**Affiliations:** School of Electrical Engineering, Vellore Institute of Technology, Vellore, Tamil Nadu, India

**Keywords:** facial emotion recognition (FER), Leaky Integrate and Fire neuron (LIF), Quadratic Integrate-and-Fire neuron (QIF), Spike-based Support Vector Machine (S-SVM), Vision Transformer (ViT)

## Abstract

Human facial emotion recognition (FER) is a vibrant research field. This research proposes a novel, biologically inspired hybrid FER framework that uniquely connects event-driven Spiking Neural Networks (SNNs) with deep learning, specifically a Spike-based Support Vector Machine (S-SVM), which is designed for its event-driven processing and energy efficiency. The article proposes a novel SNN-based framework for FER that extracts robust image features using a Vision Transformer (ViT). Spikes are generated from the features using the rate-and-threshold encoding technique. The core algorithmic novelty lies in the integration of Leaky Integrate-and-Fire (LIF) and Quadratic Integrate-and-Fire (QIF) neurons, which are used in S-SVM, creating a highly optimized decision boundary in the spike domain. The goal is to determine which neuron performs best, as confirmed by the CK+ dataset. The same technique was validated on RAF-CE, a unprocessed dataset derived from real-world events and movie scenes. The approach was validated on the CK+ dataset, achieving 99.14% and 99.94% accuracy for the QIF and LIF neurons, respectively. For the compound emotions in the unprocessed dataset, which are hard to distinguish, the accuracy rates achieved with QIF and LIF neurons are 78.87% and 98.91%, respectively. In the FER system, the LIF neuron with an S-SVM classifier performs better than the LIF neuron. This hybrid design's capacity to provide cutting-edge FER accuracy while also enabling previously unheard-of energy efficiency gains of 99.52% for CK+ and 99.60% for RAF-CE when compared to traditional deep learning models is its primary significance.

## 1 Introduction

**Figure 1:**
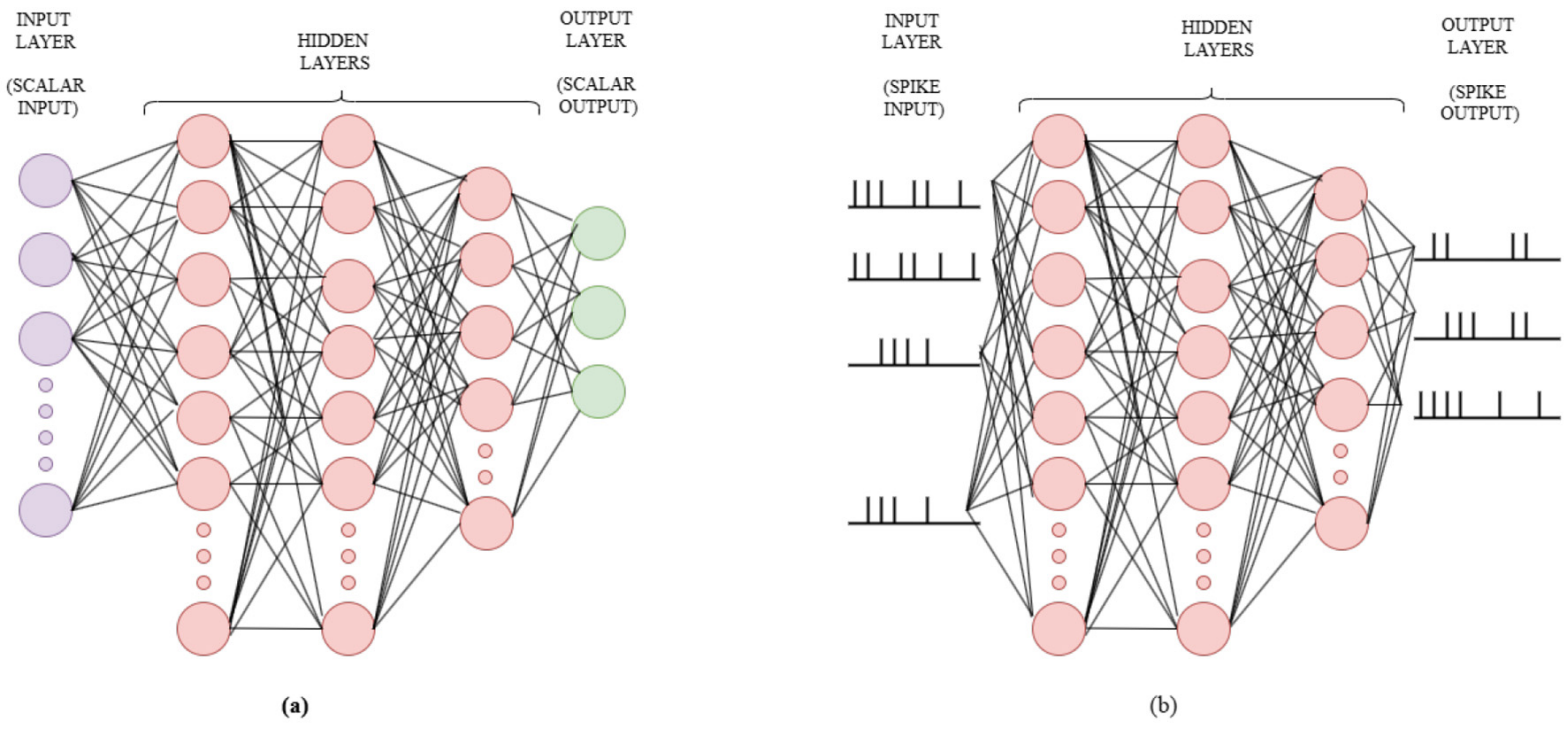
Schematic diagram of **(a)** convolutional neural network (CNN) and **(b)** spike neural network (SNN).

Facial expressions are essential to human communication because they convey emotional states instantly. By automatically identifying these intricate emotional cues, the field of facial emotion recognition (FER) has the potential to transform a wide range of cutting-edge applications. The ability to recognize these emotions automatically holds revolutionary potential for several domains, including improved driver-assistance systems, neurological disease clinical diagnoses, tailored instruction, and compassionate AI companions (Barchid et al., 2023). Convolutional neural networks (CNNs) have revolutionized the field of FER since the introduction of deep learning. They have shown unmatched capabilities in feature extraction and hierarchical representation learning, achieving state-of-the-art performance across a variety of facial expression datasets (Sajjad et al., 2023; Guo et al., 2024). Because of their intricate, multi-layered operations, which include many floating-point multiplications and accumulations, deep CNNs demand significant computing power, especially as they add more layers. Traditional ANN-based FER solutions are frequently challenging to deploy on ubiquitous edge devices, such as wearables, smartphones, or small embedded systems, where power budgets are severely constrained, and real-time processing without external cloud access is essential, because of this computational load (Roy et al., 2019). As a consequence, optimizing energy efficiency is a rigorous operational necessity for real-time edge AI rather than just an architectural choice. CNNs excel at processing data that resembles a grid, like images. SNNs, on the other hand, are bio-inspired and emulate the event-driven nature of biological neurons by communicating throughout time using discrete “spikes.” The CNN takes a scalar input and operates with synchronous firing, whereas the SNN converts the input into spikes and operates with asynchronous firing. Figure 1a shows the layers of CNN, where the input and output are taken in scalar form. Figure 1b shows the layers of SNN, where the input and output are taken in spike form.

Third-generation neural networks have been developed to overcome these restrictions. In response to the limitations, SNNs, also referred to as the third generation of neural networks, have emerged as an intriguing and fundamentally distinct paradigm. To mimic the brain's extraordinarily efficient information processing, SNNs send out sparse, discontinuous events termed “spikes” across neurons instead of CNNs, which give out continuous activation values (Yamazaki et al., 2022). The event-driven, asynchronous communication of SNNs sets them apart from their CNN counterparts. Crucially, this sparse communication in which neurons “fire” and spend energy only when information needs to be transmitted offers orders of magnitude more energy efficiency when directly implemented on specialist hardware known as neuromorphic processors (Davies et al., 2018). Because of the sparse and temporal nature of SNN computations, these specialized chips are designed to deliver unprecedented power-efficiency gains.These specialized processors are built to provide previously unheard-of power-efficiency advantages due to the sparse and temporal nature of SNN calculations, radically altering the trade-off between computational precision and battery longevity in mobile edge systems. SNNs have historically faced significant training challenges, despite their advantages in biological plausibility and energy efficiency. Because spike events are inherently non-differentiable (a neuron either fires or it doesn't, a binary event), it is difficult to directly implement conventional gradient-descent-based backpropagation, the foundation of ANN and CNN training (Pfeiffer and Pfeil, 2018). However, recent groundbreaking advances in surrogate gradient learning techniques have ingeniously resolved this problem by approximating the non-differentiable spike function via a differentiable proxy (Neftci et al., 2019). Together with the development of creative SNN structures and efficient learning rules, these advancements have significantly lessened the difficulties associated with previous training.

**Figure 2:**
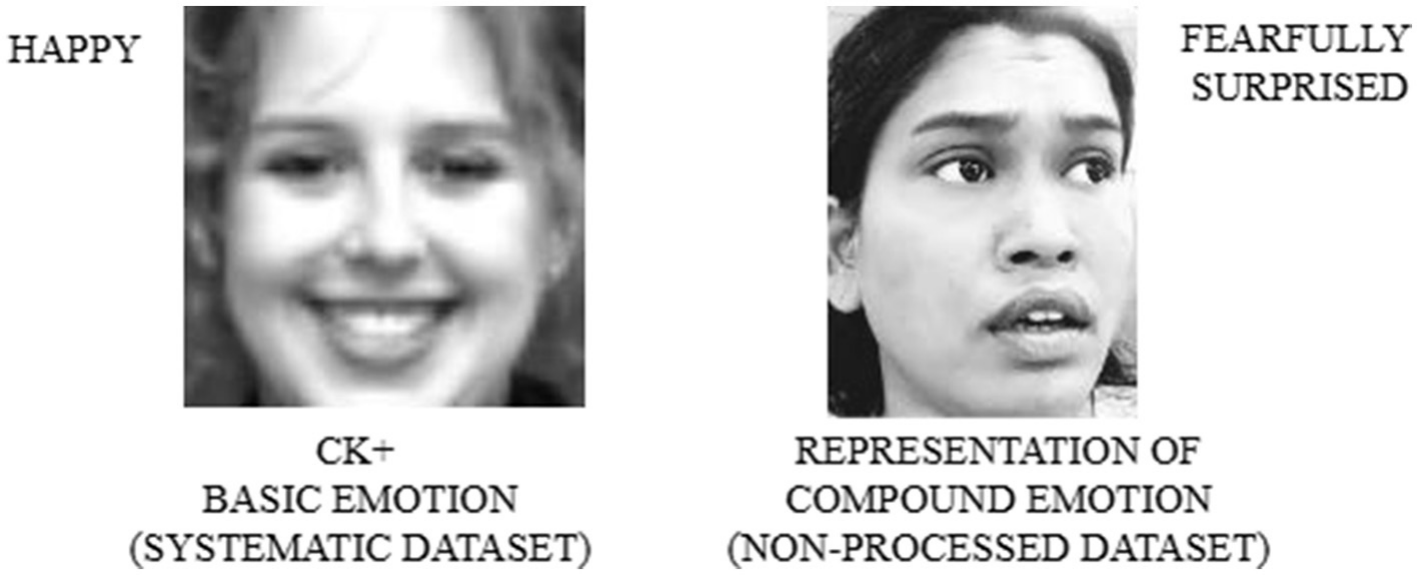
Systematic and unprocessed dataset (left image- adapted with permission from the ‘CK+ Dataset' by Lucey et al., 2010, CS229/CK+ at master · spenceryee/CS229, licensed under CC0, and right image - representation of compound emotion).

Even in low-resolution images, the high latency and power consumption of CNN architecture are significant drawbacks that this article attempts to address. The research is highly necessary because current FER models mostly rely on high-resource environments, they are unsuitable for real-time, low-power edge applications where instantaneous emotion identification could be life-saving (e.g., monitoring patient discomfort or identifying driver weariness).The FER system performs more accurately in basic emotions. There are seven different basic emotions. Compound emotion is the combination of two basic emotions. There are 21 different types of compound emotions. Compound emotions provide a much more realistic mapping of human psychology, therefore studying them is essential. However, because of their subtle, overlapping facial muscle movements, their detection adds significant complication. These subtle differences are difficult to separate using conventional feature extraction techniques, particularly when hardware constraints are present. The complexity of compound emotion recognition stems from the muscle movements involved. Both unprocessed and systematic datasets consistently demonstrate some shift in emotion recognition. Additionally, the seven fundamental emotions (such as joy, sadness, and rage) are the main emphasis of traditional FER models. However, compound emotions—blends of two fundamental states, as “happily surprised” or “angrily disgusted”—dominate human interactions in real life. Realistic affective computing requires the recognition of complex expressions, yet doing so presents significant technological difficulties. The limits of compound emotions are quite non-linear and readily blurred because they entail modest, overlapping facial muscle movements. While deep learning models demand enormous, “always-on” processing power to handle these minute changes, traditional feature extraction techniques are unable to isolate them in unconstrained situations, making edge deployment unfeasible. Additionally, the clear environmental change between controlled and uncontrolled data makes this investigation necessary. The primary distinction between the systematic and unprocessed datasets is depicted in Figure 2. Systematic datasets are collected in a laboratory environment, whereas unprocessed datasets are extracted from natural event-driven and movie scenes. In unprocessed datasets, traditional feature extraction methods like FACs and AUs are difficult to apply, as feature extraction mostly relies on images, which require straight images to extract features. This research fills the gap by utilizing SNNs in conjunction with pre-trained models and sophisticated feature-extraction techniques to address these issues. This work establishes a highly effective, robust framework that can identify complex compound emotions in real-world, unconstrained situations without falling to the latency and power costs of typical deep learning by assessing both systematic and unprocessed information.

This research work effectively bridge the gap between ultra-low-power neuromorphic classification for complex compound facial emotions and high-fidelity spatial feature extraction. Although there are hybrid models, they frequently fail when exposed to real-world domain shifts or experience information loss during spike encoding. The study presents a novel framework that combines an event-driven SNN classifier with a pre-trained ViT for reliable, structural spatial feature extraction. The classic SNN issue of processing complicated spatial patterns is overcome by this architecture, which guarantees that minor facial muscle fluctuations are caught before being processed in the resource-efficient spike domain. It directly combine LIF and QIF neurons with a novel S-SVM core classifier, in contrast to conventional SNNs that only use fully connected spiking layers. In order to assess sparse, binary spike trains, this produces a highly optimized decision boundary. Additionally, the work was validated on both highly unpredictable, unprocessed real-world data (RAF-CE) and structured laboratory data (CK+) to specifically address the performance decreases typical in uncontrolled conditions. Our hybrid model offers a remarkable 99.60% increase in energy efficiency while achieving competitive state-of-the-art accuracy (up to 99.94% on CK+ and 98.91% on RAF-CE).

The primary objective of this work is to develop a dual-optimized FER framework based on Spiking Neural Networks (SNNs) that simultaneously achieves high power efficiency and ultra-low latency, making it feasible for systems with limited energy resources without sacrificing accuracy. This work specifically focuses on creating a hybrid architecture that combines the event-driven, energy-efficient processing of SNNs using surrogate gradient learning with the strong spatial feature-extraction capabilities of pre-trained networks. By recording minute facial muscle movements without increasing the computing budget, the framework seeks to close the performance gap between basic and complex emotions. By assessing the model's performance on both structured laboratory datasets and unpredictable, unprocessed real-world data, this study also attempts to validate the model's resilience against environmental domain shifts. In the end, this study establishes energy efficiency as a key success criterion and offers a practical guide for implementing dependable, sustainable, and real-time emotion identification systems on resource-constrained edge devices and neuromorphic hardware.

## 2 Literature review

### 2.1 Theoretical foundations of facial affect: basic vs. compound emotions

Ekman's Discrete Emotion Theory, which holds that human evolutionary survival produced a collection of universally recognized expressions, is a major component of automated facial affect analysis. There are seven basic emotions: happy, fear, sad, neutral, disgust, surprise, and anger. Basic emotions are easy to recognize in a variety of models and datasets, but they have a number of drawbacks for real-world applications because they are unable to represent the complex affective states found in regular human social interactions. Early techniques mapped precise pairings of AUs to particular compound states using the Facial Action Coding System (FACS). Although these models work well in controlled lab settings, they are unable to accurately represent tiny muscle movements in real-world domain shifts, head positions, or changes in illumination. Karolinska Directed Emotional Faces (KDEF) and Japanese Female Facial Emotion (JAFFE) are two notable datasets. To find the core emotions in this dataset, Akhand et al. used a transfer-learning model, achieving 99.52% and 96.51% accuracy. The limitations of the approach are its inability to adapt to natural or unprocessed data and its lack of deployability on wearable devices. The model is easy to train, but its inference latency and computational cost are significant (Akhand et al., 2021).

Popular compound emotion datasets are Real World Affective database (RAF-DB) and Multi-Emotion Facial Expression Dataset (iCV-MEFED). While some of the compound datasets are driven by natural events, others are driven by systematic factors. Appasaheb Borgalli et al. report a 74% misclassification rate and 26% accuracy using the iCV-MEFED dataset, Multi-Class Support Vector Machine (mSVM) as the classifier, and Inceptionv3 as the feature extractor. This sharp decline in performance demonstrates how conventional deep structures fall short when handling fine-grained affective boundaries. The computational cost of fine-grained categorization, the difficulty of extracting data such as minute muscle movements, and the method's incapacity to precisely detect complex compound emotions are some of its drawbacks (Borgalli and Surve, 2023).

### 2.2 Feature drift in unprocessed datasets: a problem

Unprocessed data shows a change in accuracy due to feature extraction from images. In Yu and Lu (2025) utilize Qwen-vl as the model for compound emotion recognition and primarily concentrate on aligned datasets. The model's accuracy for compound emotion is 78.50%, and for basic emotion, it is 89.78%. The aligned dataset, which highlights the faces being recognized. Using higher-resolution images (448*448) is challenging for this model and incurs significant computational expense. This disparity in performance highlights how compound emotions significantly change the way facial landmarks are arranged structurally. Muscle movements make it difficult to recognize compound emotions because, the Facial Action Coding System (FACS) states that different muscle movements known as Action Units (AUs) create expressions. Due to a shift in Action Units (AUs), certain compound emotions are misclassified and resemble other compound emotions. Individual AUs are difficult to identify and isolate statistically when the facial features are pulled in opposite ways, creating delicate blends where micro-expressions overlap (Gupta et al., 2023; Wagner, 2019).

### 2.3 Assessment of handcrafted feature extractors and face detection

MTCNN, RetinaFace, and Viola-Jones are a few techniques for initial face detection. In this, MTCNN and Retinaface fall under the category of automated feature extraction methods. Whereas bounding boxes are utilized to locate and extract features using the Viola-Jones method. In the article, Nivodhini et al. (2025) used an algorithm for identifying emotions and face detection, showing an accuracy of 81%. The technique's primary flaw is that it can only perform binary classification, regardless of whether faces are present, indicating that it is more of a localization tool than a thorough emotional feature analyzer. Its drawbacks include limited feature richness for emotion analysis, sensitivity to occlusion, and fixed-size window detection, making it difficult to use with real datasets. The MTCNN and Retinaface algorithms simplify face alignment, making the task easier. In addition to making detection and classification easier, it crops the pictures from the source image. According to Onaran et al. (2024), high-quality evaluations are generally associated with superior ability in identifying faces and facial expressions, respectively. The features of the identified faces are extracted using a variety of methods, ranging from conventional to pre-trained CNN models. Local binary patterns (LBP), histogram of oriented gradients (HOG), and geometric feature extraction are three traditional feature extraction methods. From the LBP features, handcrafted features are extracted. It facilitates feature extraction in systematic data but complicates it in unprocessed data. Using SVM as the classifier, C. Shan et al. show that LPB performs well across a range of systematic datasets. While the JAFFE dataset has a 79.8% MMI, the MMI was 86.7% (Shan et al., 2009). The limitations are mostly due to the inability to fully capture the intricacy of dynamic, unrestricted, and real-world facial emotions, as well as the intrinsic limitations of handcrafted features compared to deep network-trained features. Crucially, the complete lack of temporal information, which treats human feeling as a collection of disjointed, static frames, greatly restricts this LBP feature extraction. This LBP feature extraction is further limited by the absence of temporal information. J. Chen et al. show 94.3% and 88.7% accuracy for the systematic datasets CK+ and JAFFE using SVM as the classifier (Chen et al., 2014). Although it exhibits good accuracy, it suffers significantly from out-of-plane head rotations (e.g., in profile views) and generates substantial rotational invariance within the image plane (due to orientation bins). The effectiveness of HOG is diminished since head posture significantly alters the look of facial components. In “unprocessed” datasets that are highly complex or diverse, it cannot automatically learn more abstract, hierarchical, or context-aware features that might be more suitable. For compound emotions, it also lacks time information.

### 2.4 Modern architectural evolutions in CNN-based FER

Modern CNN configurations have taken over the FER sector in an effort to get around the strict limitations of customized pipelines. Standard deep learning models, such as VGG16, ResNet, and basic Vision Transformers (ViTs), are computationally expensive for real-time, resource-constrained edge systems because they scale up parameter counts unsustainably. Recent advances have significantly shifted toward structural optimization and targeted face perception mechanisms to address this constraint. Gursesli et al. (2024) presented a Custom Lightweight CNN-based Model (CLCM) suited for edge deployment in response to the computational drawbacks of heavy architectures (Gursesli et al., 2024). Their architecture showed that a lightweight footprint may achieve very competitive accuracies of 84% on RAF-DB and 63% on FER-2013, beating larger networks like MobileNetV2, by reducing the parameter footprint of conventional networks to 2.3 million parameters (Gursesli et al., 2024). Although this structural compression lowers inference latency and makes real-time edge processing easier, lightweight models' ability to simulate abstract feature interactions is intrinsically compromised, and they often show performance degradation when exposed to extreme head poses or severe in-the-wild facial occlusions.

Simultaneously, recent developments have concentrated on improving the quality of structural spatial features by switching from uniform holistic tracking to multi-domain facial segmentation. In order to precisely address this, Tian et al. (2025) suggested a Perception CNN (PCNN) architecture that is intended to maintain localized facial micro-structures under difficult variations (Tian et al., 2025). Their model makes use of five parallel sub-networks designed to capture small differences across different sense organs, such as the mouth, cheeks, and eyes, and uses a multi-domain interaction and registration method to map these changes back to a global structural skeleton (Tian et al., 2025). The model achieves state-of-the-art benchmarks in both lab settings and real-world datasets thanks to this particular fine-grained extraction, which enables the model to maintain crucial landmark associations even during extreme head posture adjustments or self-occlusions (Tian et al., 2025).

### 2.5 Comparing spiking neural networks and deep learning paradigms

In addition to traditional feature extraction, CNN-based methods such as EfficientNet, VGGNet, and ResNet are used. CNN-based feature extractors offer higher-quality features. Since VGG16 has more layers, it is computationally expensive to train and infer. It is challenging to identify features in a unprocessed dataset. The feature extraction procedure is redundant. In this article, more features are extracted. A. K. Dubey et al. accurately depict the core emotions of CK+ and JAFFE (Dubey and Jain, 2020). The layers employed also affect the other CNN-based feature extractors. When the image size is frozen to a big value using a CNN-based feature extractor or ViT, training and optimization become extremely difficult, leading to severe hardware constraints. Training becomes more complicated when the image size is frozen to a large value using a CNN-based feature extractor or transformer. The accuracy values of Xception Net, Inception Net, ResNet (with different numbers of layers), Recurrent Neural Network (RNN), and Multi-Layer Perceptron (MLP) have varied across datasets. Using these different classifiers (for static ANNs) results in high energy consumption, a lack of temporal dynamics, excessive reliance on big datasets for generalization and inefficient processing of temporal input. In the end, employing these many classifiers for static ANNs leads to excessively high energy consumption, a complete lack of temporal dynamics, an over-reliance on large datasets for generalization, and extremely inefficient sequential data processing.

As the third generation of ANNs, SNNs offer a paradigm shift to get beyond these computational obstacles. Despite their exceptional accuracy, the Spike neural network, the third generation of ANNs, uses a tremendous amount of energy during training and testing (Maass, 1997). Compared with the most advanced models, SNN will exhibit lower accuracy. The model can be implemented using SNN on wearable technologies and other devices with the help of a neuromorphic chip. S. Barchid et al. show that SNNs are 47.42–to 65.39– more energy-efficient than comparable artificial neural networks (Barchid et al., 2023). Neurons only compute and transmit information (spikes) when their membrane potential surpasses a threshold, in contrast to ANNs, which continually assess real-valued activations. Consequently, computation becomes asynchronous and sparse. Crucially, SNNs are inherently equipped with rich spatial-temporal dynamics because they naturally encode data in both precise time and spike rate, perfectly matching the fluid, dynamic nature of moving human facial expressions. The type of image or video determines how the spikes are generated. G. Pu et al. show an accuracy for the FER+ and FER 2013 datasets as 79.97% and 61.87%, respectively (Pu and Chen, 2024), demonstrating that in real-time edge contexts, SNNs provide a practical, low-power method for resolving complex compound emotions.

The proposed study overcomes the aforementioned constraints by using a FER technique. The FER system's primary shortcoming is that, due to its complex features, it cannot recognize compound emotions. The proposed study uses transformers as a feature extractor. The ViT treats an image as a sequence of words, in contrast to conventional CNNs, which process local pixels (edges/textures). The spikes are generated, and the energy-efficient SNN classifier is used to classify the emotions. Low latency, high battery consumption, computational expense, and difficulty are further disadvantages that can be overcome. The rest of this article is structured as follows: Section 3 describes the proposed method and provides a brief overview of the strategy used. It also lists the datasets that were used to evaluate the proposed system. Section 4 covers the implementation of the work. Section 5 presents the results and future development of the proposed method.

## 3 Proposed methodology

**Figure 3:**
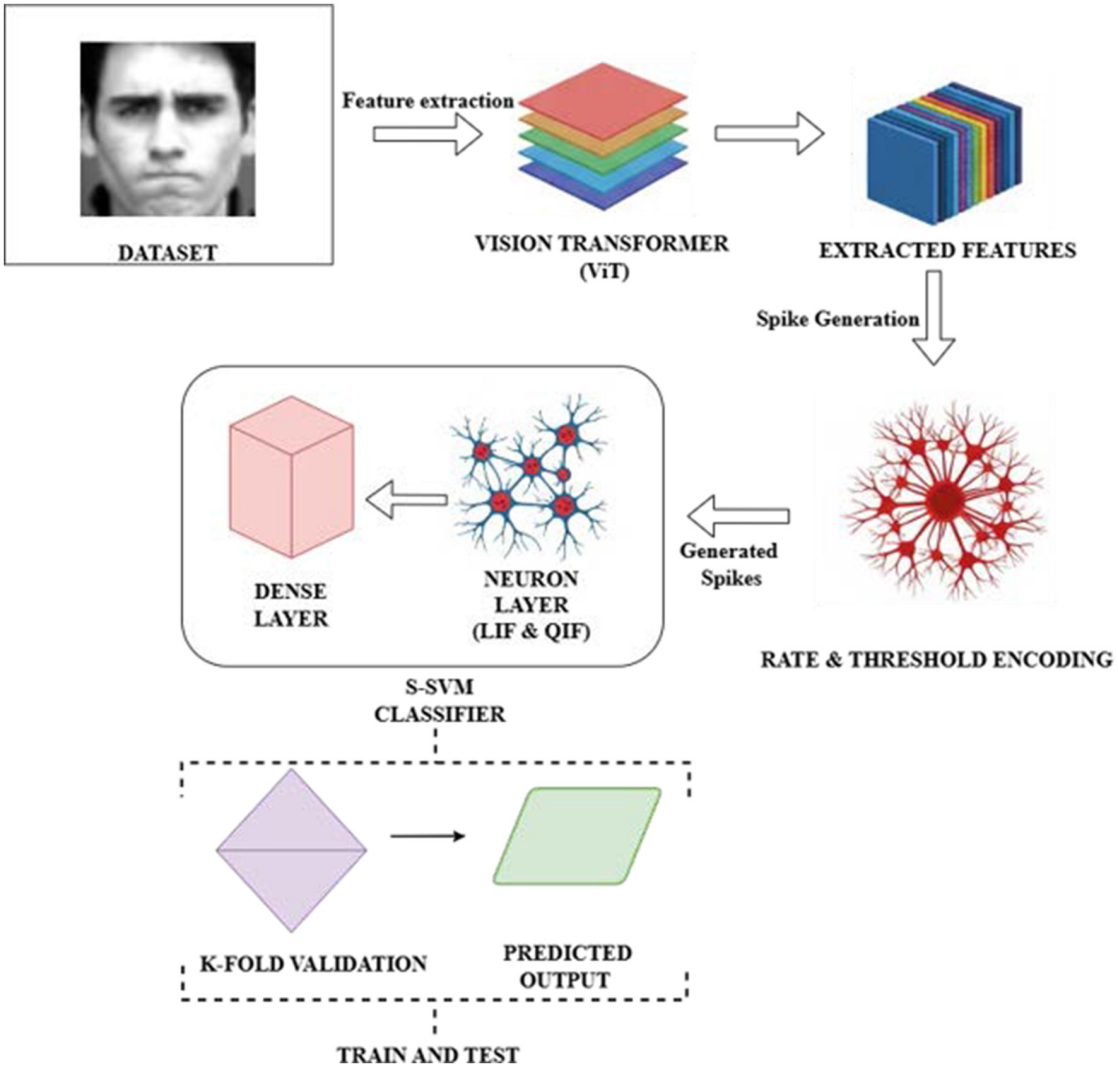
Proposed methodology (the facial image has been adapted with permission from the ‘CK+ Dataset' by Lucey et al., 2010, CS229/CK+ at master · spenceryee/CS229, licensed under CC0).

The proposed model presents a new information-preserving pipeline that dynamically connects temporal, event-driven computing with deep spatial representations. The system uses a high-capacity Transformer-based feature extractor instead of conventional convolutional backbones to capture global context and long-range dependencies across complicated facial geometries—features that are usually lost in shallow spiking architectures. Our unique spike-generation interface is a significant methodological innovation. A calibrated rate-and-threshold encoding technique is used to convert the acquired high-dimensional spatial data into discrete, sparse temporal spike trains, therefore condensing the continuous feature space without sacrificing semantic correctness. A new neuromorphic classification layer receives these temporal representations after that. Our architecture directly combines the dynamics of LIF and QIF neurons with a Spike-based Support Vector Machine (S-SVM) core, as opposed to typical, fully linked spiking layers that are vulnerable to optimization drift. Using the maximum-margin separation principles of SVMs and the biological efficiency of SNNs, this hybrid classification engine creates extremely reliable decision boundaries in the spike domain. Lastly, the network's structural hyperparameters are rigorously tested using a *K*-fold cross-validation approach and globally optimized to guarantee stringent generalization against severe environmental domain alterations. The suggested model is depicted graphically in Figure 3.

### 3.1 Dataset

We used two datasets in the approach one for basic emotion recognition and the other for compound emotion recognition. A systematic dataset of basic expressions is utilized to validate the suggested approach, and the algorithm is then applied to the “unprocessed” dataset of compound emotions. Extended Cohn-Kanade (CK+) (Lucey et al., 2010) this is the basic dataset of emotions that our approach uses. Since CK+ consists primarily of posed, peak expressions, it offers a controlled environment for studying particular basic emotions. It is, however, not very applicable to spontaneous expressions, which often contain complex cues and emotional combinations. A total of 123 people are involved in documenting the emotion. The dataset includes 981 images with 7 emotions: anger, contempt, disgust, fear, happiness, sadness, and surprise.

The Real-world Affective Faces dataset of Compound Emotions (RAF-CE) (Li et al., 2023). It is an additional dataset we used in our methodology. But RAF-CE provides a more realistic representation of human affect with nuanced emotions (such as “happily surprised”) and facial expressions captured in unconstrained real-world scenarios. Variations in head posture, illumination, occlusions, and individual differences in expressive behavior significantly increase the recognition challenge. There are 10 compound emotion classes in the dataset, which include 3,806 images: Happily Surprised, Happily Disgusted, Sadly Fearful, Sadly Surprised, Sadly Disgusted, Fearfully Angry, Angrily Surprised, Fearfully Surprised, Sadly Angry, Angrily Disgusted. From the total dataset, 80% is used as the training dataset and 20% as the testing dataset. The features are then extracted from the dataset.

### 3.2 Feature extraction using ViT

**Figure 4:**
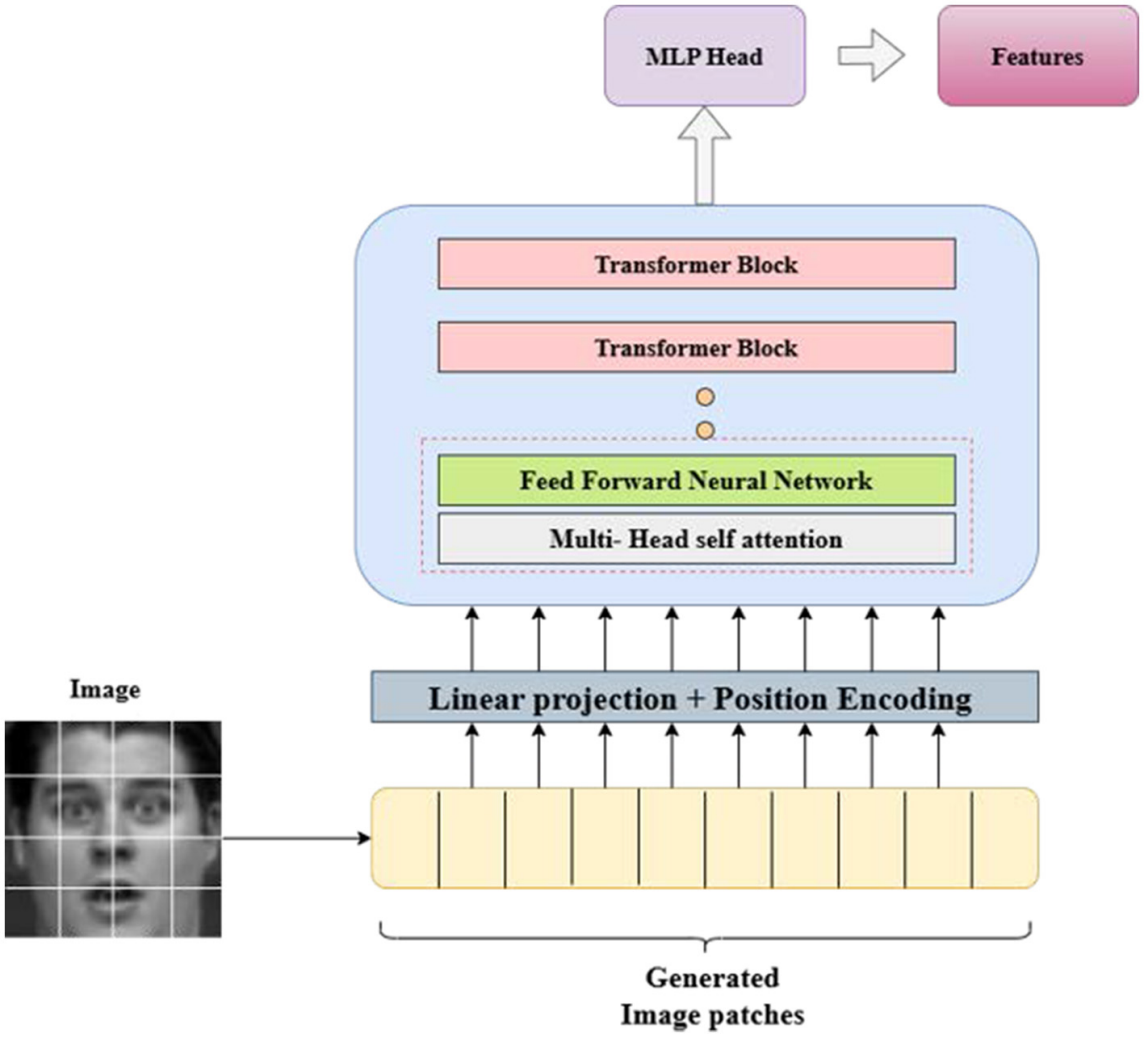
Vision transformer architecture (the facial image has been adapted with permission from the ‘CK+ Dataset' by Lucey et al., 2010, CS229/CK+ at master · spenceryee/CS229, licensed under CC0).

Similar to how Large Language Models handle words, the ViT reimagines visual data as a series of discrete patches, serving as a potent feature extractor. This framework breaks down an input image into square patches of a predefined size, usually 16 by 16 pixels. These patches are then flattened and mapped onto a high-dimensional embedding space. To aggregate data throughout the entire image, a distinct Classification (CLS) token is appended to this sequence. As these tokens pass through multiple layers of the Transformer encoder, the Self-Attention mechanism calculates the relevance of every patch to every other patch, regardless of their spatial distance. This enables the model to capture intricate structural links and global dependencies that conventional CNNs would otherwise overlook due to their limited receptive fields. The last Multi-Layer Perceptron (MLP) or “head is eliminated to use ViT only for feature extraction.” Rather, the raw vector output of the last encoder layer is extracted. This resulting latent representation provides a detailed, semantic summary of the image's visual content. Figure 4 shows the ViT architecture for feature extraction.

These rich feature vectors are quite flexible; they can be incorporated into multimodal architectures that connect computer vision and natural language processing, fed into simpler classifiers, or used for similarity searches in image retrieval systems. ViT offers a “universal” visual backbone that is excellent at recognizing both fine-grained elements and overall scene context by utilizing pre-trained weights from enormous datasets (Dosovitskiy et al., 2021).

### 3.3 SMOTE

A powerful statistical method for addressing class imbalance in datasets where one class (the minority) is notably underrepresented relative to others is Synthetic Minority Over-sampling Technique (SMOTE). Deep architectures like ViTs can easily overfit to particular background or illumination artifacts because traditional over-sampling techniques often rely on the simple reproduction of existing data points. In contrast, SMOTE expands the “decision region” of the underrepresented class by interpolating across existing minority examples, so preventing this memorizing. The method selects an instance of a minority class and finds its k-nearest neighbors in the same class (Chawla et al., 2002). After that, it randomly selects one of these neighbors and adds a new data point at a random location along the line segment that joins them. The sample s is created using the formula shown Equation 1:


$$\begin{array}{l} s=x_{i}+\text{rand}\left(0,1\right)\times \left(x_{zi}-x_{i}\right) \end{array}$$ (1)

Where *x*_*i*_ is the original point, *x*_*zi*_ is the selector neighbor, and *s* is the synthetic point created between the minority sample *x*_*i*_ and the selector neighbor *x*_*zi*_.

The hybrid spatial-temporal pipeline places SMOTE in a way that maintains neuromorphic integrity and avoids performance loss. The data augmentation is carried out strictly within the continuous high-dimensional embedding space of the ViT before the spike-generation phase, instead of applying SMOTE downstream to discrete binary spike trains, which would produce mathematically invalid fractional spikes and interfere with the temporal dynamics of the LIF and QIF neurons. The rate-and-threshold encoding layer that follows can convert the continuous feature vectors into legitimate, event-driven temporal spike trains by first balancing them. Therefore, SMOTE's application within the pre-spike continuous feature domain remains a highly effective tool for resolving the severe class imbalances typical of complex compound emotions, despite its known theoretical drawbacks, such as ignoring the majority class density, which can occasionally cause “bridging” or class overlapping (Han et al., 2005).

### 3.4 Spike generation

**Figure 5:**
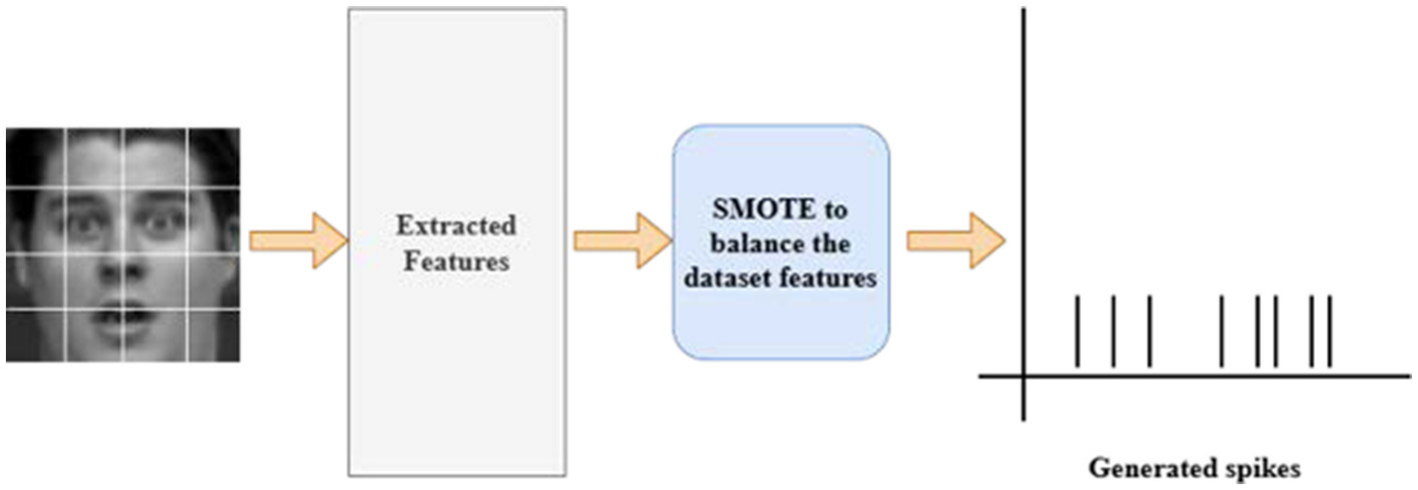
Spike generation (the facial image has been adapted with permission from the ‘CK+ Dataset' by Lucey et al., 2010, CS229/CK+ at master · spenceryee/CS229, licensed under CC0).

Spike is created using attributes extracted from the previously processed and augmented images. Unlike ANNs, which employ continuous values, SNNs mimic the human brain by communicating through discrete, time-sensitive events called spikes. Because spikes are the native language of energy-efficient neuromorphic technology, SNNs perform incredibly well on specialized processors. Because spikes occur at specific times, SNNs are inherently capable of handling temporal information. Spikes enable computing using events. Neurons “fire” and emit a spike when necessary; otherwise, they remain dormant. This results in significantly lower energy consumption than ANNs. In contrast to CNNs' real-valued, multi-bit activations, SNNs transmit simple binary spikes, which minimize complex arithmetic and data movement. Unlike CNNs, which do dense, continuous computations, SNNs only activate neurons in response to spikes. Significant energy savings are achieved by this “activity-dependent” operation. There are different types of spike generation, like rate encoding, threshold encoding, Population encoding, Poisson encoding, and time-to-first-spike (TTFS) encoding. The rate-encoding and threshold-encoding techniques have been applied in our study model. Figure 5 shows the spike generation from the balanced SMOTE features.

#### 3.4.1 Rate encoding

Static features (ViT embeddings) are first transformed into a continuous input current. The input current *I* injected into the SNN at each time step *t* for an input vector *x* is defined by Equation 2:


$$\begin{array}{l} I\left(t\right)=W.x+b \end{array}$$ (2)

The current stays constant during the simulation window since the input *x* is non-temporal (a static image feature) as shown in Equation 3:


$$\begin{array}{l} I^{\left(1\right)}=I^{\left(2\right)}=\cdots =I^{\left(T\right)} \end{array}$$ (3)

The LIF neuron is where the majority of encoding occurs. Over time, this current is accumulated by the membrane potential U. The discrete- time update for the ith neuron shown in Equation 4:


$$\begin{array}{l} U_{i}\left[t+1\right]=\beta U_{i}\left[t\right]+I_{i}\left[t+1\right]-S_{i}\left[t\right]\theta \end{array}$$ (4)

Where

β (decay) is the leak factor (0.95), representing the membrane time constant,

*I* (input) is the steady current derived from the ViT features, and

*S*[*t*]θ (reset) denotes the “Reset-after-spike” method, where the potential is lowered by the threshold value θ if the neuron fired at the previous step.

The spike is generated as a binary output based on the Heavisine step function H(.) as shown in Equation 5:


$$\begin{array}{l} S_{i}\left[t\right]=H\left(U_{i}\left[t\right]-\theta \right) \end{array}$$ (5)

where *S*_*i*_[*t*] ϵ 0, 1.This creates a sparse, 50-step binary temporal sequence from the continuous feature space. It is necessary to collapse the temporal dimension to interface the SNN with the SVM. We use Rate Encoding, which assumes that “firing intensity” rather than the precise timing of individual spikes—carries the information. The total spike count *C*_*i*_ for the i-th neuron is:


$$\begin{array}{l} C_{i}=\overset{T}{\underset{i=1}{\sum }}S_{i}\left[t\right] \end{array}$$ (6)

Equation 6 shows how the spikes are generated using the rate-encoding technique.

#### 3.4.2 Threshold encoding

The non-linear process known as threshold encoding transforms a continuous build-up of “evidence” (the membrane potential) into a binary, discontinuous output (the spike). The threshold serves as the information gatekeeper, while the weights and biases define the signal's strength. The neuron must integrate data over time before encoding a spike. A neuron's membrane potential U is a representation of its state as shown in Equation 7.


$$\begin{array}{l} U\left[t+1\right]=\beta U\left[t\right]+I\left[t+1\right] \end{array}$$ (7)

Where,

β is the decay rate (0.95); the “leakiness” of a biological membrane is simulated.

I[t+1] is the input current derived from ViT features Wx+b.

When the membrane potential U approaches or exceeds a predetermined value theta, the “Encoding” event occurs. The output spike is a binary value as shown in Equation 8 and the condition for S[t+1] is shown in Equation 9.


$$\begin{array}{l} S\left[t+1\right]=H\left(U\left[t+1\right]-\theta \right) \end{array}$$ (8)

where:


$$\begin{array}{l} S\left[t+1\right]=\{\begin{array}{c} 1\;\text{if }U\left[t+1\right]\geq 0\\ 0\;\text{if }U\left[t+1\right]>0 \end{array} \end{array}$$ (9)

The “analog” data from the ViT is converted into a temporal “spike” at this stage. A neuron must be reset to prevent it from continuously firing after it crosses the threshold and encodes a spike. By using Reset by Subtraction, the membrane potential is decreased by the threshold value as shown in Equation 10


$$\begin{array}{l} U_{next}=U\left[t+1\right]-S\left[t+1\right].\theta \end{array}$$ (10)

In the event of a spike (S = 1), the potential decreases by θ. For the following time step, the potential stays the same if there was no spike (S = 0). For a steady input, the potential U grows toward a steady state of $\frac{I}{1-\beta }$. A neuron will only encode information if: $\frac{I}{1-\beta }>0$. For the neuron to fire with the settings (θ = 1.0, β = 0.95), the input current must be greater than 0.05.

### 3.5 S-SVM classifier

The hybrid classification system known as S-SVM unites the statistical robustness of Support Vector Machines with the temporal, bio-inspired dynamics of SNNs. A S-SVM is an example of how event-driven neuromorphic computing has replaced conventional vector-based computation. A conventional SVM finds a hyperplane that optimizes the “margin” or gap between several classes by treating the data as static points in a multi-dimensional space. However, this spatial geometry is translated into the temporal domain by a spike-based SVM. The technology uses discrete “spikes” or pulses to communicate rather than analyzing raw numbers. The model can leverage the exceptional energy efficiency of the human brain because input features are stored as spike trains, in which information is carried either by pulse frequency (rate coding) or by the precise timing of a single pulse (temporal coding).

A spiking neuron model typically the LIF neuron or the QIF neuron performs the actual categorization. This neuron serves as the biological counterpart of the SVM's decision boundary. When spikes reach the neuron, they are integrated into the neuron's “membrane potential” after being multiplied by synaptic weights, which match the support vector coefficients. The neuron fires an output spike, indicating that the input belongs to a given class, if the potential exceeds a threshold (representing the SVM's bias). Because it is event-driven, the system is “asynchronous” and consumes only power during spikes, making it ideal for battery-constrained applications such as wearable medical devices or edge sensors.

**Figure 6:**
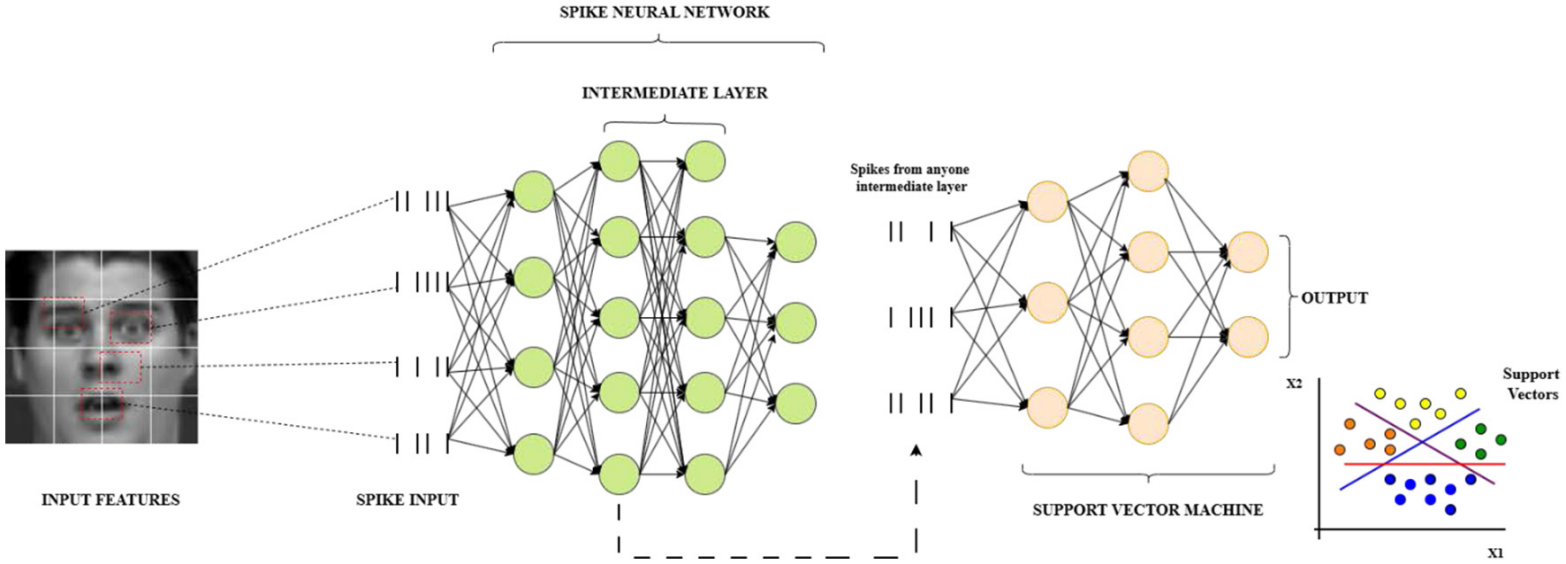
S-SVM architecture (the facial image adapted with permission from the ‘CK+ Dataset' by Lucey et al., 2010, CS229/CK+ at master · spenceryee/CS229, licensed under CC0).

The non-differentiable spiking process is the main obstacle to creating these models. Since a spike is a binary event that is “all-or-nothing,” its mathematical derivative is 0 everywhere except at the precise instant of firing, when it is undefined. This makes it impossible to employ ordinary backpropagation, which is the foundation of contemporary AI. For the sole purpose of computing gradients, a smooth, continuous curve (such as a Sigmoid or Gaussian function) is substituted for the sharp “step” of the spike during the training phase. This enables the model to retain its discrete, spike-based behavior during actual deployment while being trained using conventional gradient descent. Figure 6 shows the S-SVM architecture.

Finally, the high-speed, low-power performance of neuromorphic hardware is provided by a spike-based SVM trained using surrogate gradients. A spike-based SVM may frequently make a classification conclusion as soon as the first few important pulses arrive, in contrast to conventional frame-based systems that have to wait for the collection of the entire set of data. Because of this, the technology is especially powerful for real-time applications, including processing data from “event-based” silicon retinas, high-speed robots, and gesture recognition.

ALGORITHM:

STEP 1: Define the surrogate Gradient function: Construct a custom function (surrogate gradient fast sigmoid) with a smooth, differentiable approximation of the gradient for the backward pass and a hard threshold (if spike input > 0) for the forward pass.

STEP 2: Create a custom LIF or QIF neuron layer:

- Set up synaptic weights and neuron characteristics (threshold, reset voltage, dynamics coefficients, etc.).
- In the forward pass call method:
  - Update neuron membrane potentials according to LIF or QIF dynamics and input for every timestep.
  - Use the specified surrogate gradient function to ascertain whether a spike (1 or 0) takes place.
  - Reset the membrane potential and accumulate the spike if one happens.
- Give back each neuron's total accumulated spikes.

The dynamics for LIF and QIF are shown below with a mathematical equation.

#### 3.5.1 LIF (Leaky Integrate and Fire) neuron dynamics

The LIF neuron strikes a good balance between biological realism and simplicity, making it suitable for large-scale simulations. We describe the mathematical model for our suggested method. In our study, the LIF neuron model describes how a neuron's membrane potential (V) varies over time in response to an incoming current (I). Equation 11 illustrates how the input current *I*_*n, i*_(*t*) for each neuron i in the LIF layer is calculated as a weighted sum of the incoming spikes from the previous layer at each time step t:


$$\begin{array}{l} I_{n,i}\left(t\right)=\underset{j}{\sum }S_{n,j}\left(t\right)\cdot w_{j,i} \end{array}$$ (11)

Where *S*_*n, j*_(*t*) is the spike from input feature j at time t for sample n, *w*_*j, i*_ is the Synaptic weight connecting input feature j to LIF neuron i.

When the neuron receives the input current, the membrane potential will alter. The membrane potential change is depicted in Equation 12, a simplified mathematically calculated equation.


$$\begin{array}{l} V\left(t+1\right)=V\left(t\right)\cdot \left(1-\frac{dt}{\tau }\right)+I_{n,i}\left(t\right) \end{array}$$ (12)

where V(t) is the initial membrane potential, τ is the membrane time constant, *I*_*n, i*_(*t*) is the input current, and dt is the simulation time step duration. A spike is produced when the membrane potential surpasses the threshold voltage (*V*_*threshold*_), as Equation 13 illustrates:


$$\begin{array}{l} S_{n,i}\left(t+dt\right)=V\left(t+dt\right)-V_{\text{threshold}} \end{array}$$ (13)

The membrane potential is reset using *V*_*reset*_, which is indicated in Equation 14, once the spike has been formed. Equation 15 displays all of the spikes that have been accumulated over all time steps.


$$\begin{array}{l} V\left(t+dt\right)=V_{reset} \end{array}$$ (14)

$$\begin{array}{l} \text{Output}=\overset{T-1}{\underset{t=0}{\sum }}S_{n,i}\left(t\right) \end{array}$$ (15)

The network can learn thanks to the smooth, non-zero gradient signal it delivers. In the final output layer, the categorization decision is based on the spiking activity of the output neurons. The following parameters are hyperparameter tuned: *V*_*threshold*_, *V*_*reset*_, τ, V(t), and dt.

#### 3.5.2 QIF (Quadratic Integrate and Fire) neuron dynamics

Quadratic Integrate-and-Fire (QIF) neurons are a model of spiking neurons that describes the “Type I excitability” that certain genuine neurons display, enabling them to fire at arbitrary low frequencies around their firing threshold. Equations represent the mathematical model for our suggested method. As demonstrated in Equation 11, the input current is produced when the spikes from rate encoding enter the QIF neuron, much like in the LIF neuron. Equation 16 illustrates how the membrane potential alone differs from LIF neurons.


$$\begin{array}{r} V\left(t+1\right)=V\left(t\right)+\Delta t\cdot [-g_{L}\left(V\left(t\right)-E_{L}\right)+I_{n,i}(t)\\ +q\cdot {(V(t)-V_{\text{th\_fire}})}^{2}] \end{array}$$ (16)

When the membrane potential is updated, the spikes are produced as shown by Equation 13, where Δ*t* is the time step duration, EL is the leak reversal potential, gL is the leak conductance (direct gL determines the times constant of the leak term), *V*_*t*_*h*__*f*_*ire*_ is the effective firing threshold, and q is the quadratic coefficient.


$$\begin{array}{l} S_{n,i}\left(t+dt\right)=V\left(t+1\right)-V_{\text{th\_fire}} \end{array}$$ (17)

Equations 14 and 15 demonstrate that the total number of spikes accumulated is comparable to that of a LIF neuron and that the membrane potential is reset after spikes are formed. Similar to LIF neuron, a surrogate gradient mechanism is used to train the QIF neuron. V(t), *V*_*reset*_, gL, EL, *V*_*th*_*fire*_, Δ*t*, and q are the parameters of QIF neuron as shown in Equation 17.

## 4 Implementation

This section explains in detail about the dataset preparation and spike generation for emotion recognition classification, along with the evaluation metrics and hyperparameter settings.

### 4.1 Preprocessing steps

A basic processing sequence is used during preprocessing for the CK+ and RAF-CE datasets to convert unprocessed, diverse facial imagery into a uniform numerical representation ideal for deep learning backbones. The first step in the procedure is Color Space Alignment, which transforms the original grayscale images typical of the Cohn-Kanade dataset into a three-channel RGB format. In the RAF-CE dataset, to prevent grayscale or multi-channel transparency layers from causing dimensional mismatches during training or feature extraction, every image is explicitly converted to the RGB color space during data fetching. To ensure architectural compatibility with models like the ViT, which anticipate multi-channel inputs, the single luminance channel is replicated across the Red, Green, and Blue planes. Leverage depends on this alignment since it enables the model to use strong pre-trained spatial weights from large datasets, such as ImageNet, without requiring architectural changes, which significantly speeds up downstream convergence. The images go through a rigorous series of statistical and spatial adjustments after they are loaded. Every image is first forced into a fixed 224 × 224-pixel resolution via a resize procedure. The patch-embedding layer of the transformer architecture, which divides the global image into precisely 196 non-overlapping 16 × 16 patches for localized feature analysis, requires this particular dimensionality. By standardizing this spatial resolution, spatial variable-length batching problems are eliminated and the ViT can consistently record localized, fine-grained facial muscle contractions in a variety of people.

Tensorization and Range Scaling complete the conversion from visual to numerical input. To align the memory layout with the GPU's requirements, this phase rearranges the data layout from “Height-Width-Channel” (HWC) to “Channel-Height-Width” (CHW) and scales the pixel values from the integer range [0, 255] to the floating-point range [0.0, 1.0]. This prevents large integer values from causing numerical instability, preparing the data for gradient-based optimization. The last and most important stage is Zero-Center Normalization, which involves shifting the pixel distribution into a symmetrical [–1, 1] range by applying a mean () and standard deviation (σ) of 0.5. In order to ensure that the gradients computed during backpropagation can fluctuate optimally in both positive and negative directions, systematic bias in network activations is prevented by centering the input distribution around a zero mean. By stabilizing the loss landscape, this guarantees that the input signals have a uniform distribution, greatly reducing the possibility of vanishing or inflating gradients during training and enabling greater peak classification accuracy. To preserve numerical stability, the data must be centered around zero. This ensures the input signals have a uniform distribution, preventing gradients from disappearing or blowing up during learning. When taken together, these preparation procedures ensure the model receives clean, standardized, and mathematically optimized input for high-performance classification tasks.

### 4.2 Feature extraction

A ViT is used for feature extraction from the CK+ and RAF-CE datasets. The remarkable capacity of the ViT-Base model to extract significant, high-dimensional features from intricate facial expression datasets is shown in both t-SNE representations. The t-SNE plot of the CK+ dataset exhibits high structural clarity. Particularly for unique expressions like surprise and delight, there is a clear and promising separation across clusters. These emotions are defined by distinct geometric and textural patterns that the model has effectively discovered, resulting in dense, localized clusters. This suggests that ViT-Base features are highly reliable on controlled datasets, providing a clear “map” of human emotion that is easy for a classifier to understand.

The RAF-CE visualization, on the other hand, displays a more intricate, continuous feature space. The model's capacity to capture the subtlety and overlap present in “in-the-wild” facial emotions is highlighted by the fact that the clusters are more interleaved than in CK+. The approach acknowledges the small gradients across similar expressions rather than viewing emotions as discrete islands. A significant degree of feature diversity is suggested by the wide distribution across the plot, indicating that the ViT-Base captures a broad range of facial changes, lighting conditions, and angles—all of which are essential for real-world generalization.

**Figure 7:**
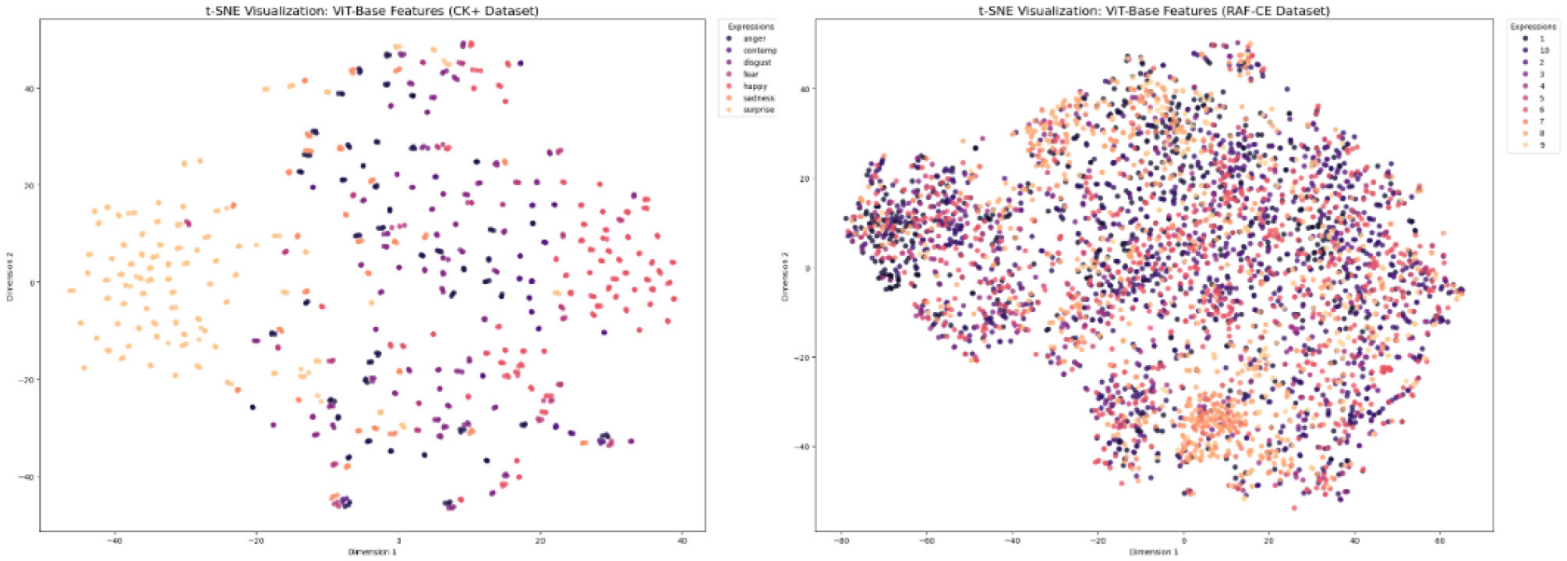
t-SNE visualization of CK+ and RAF-CE dataset extracted features.

Figure 7 shows the t-SNE visualization of the features extracted from both the CK+ and RAF-CE datasets using ViT. It is clear from combining the two plots that the ViT-Base design is very adaptable. While retaining a comprehensive and representative perspective of more varied, naturalistic data (RAF-CE), it excels at identifying precise, discriminative boundaries in cleaner contexts (CK+). The model's learned representations are dependable and grounded in real visual cues of expressions across various data distributions, as evidenced by the constancy in color distributions, where comparable colors still tend to drift toward specific locations in both plots.

### 4.3 SMOTE applied to the extracted features

**Figure 8:**
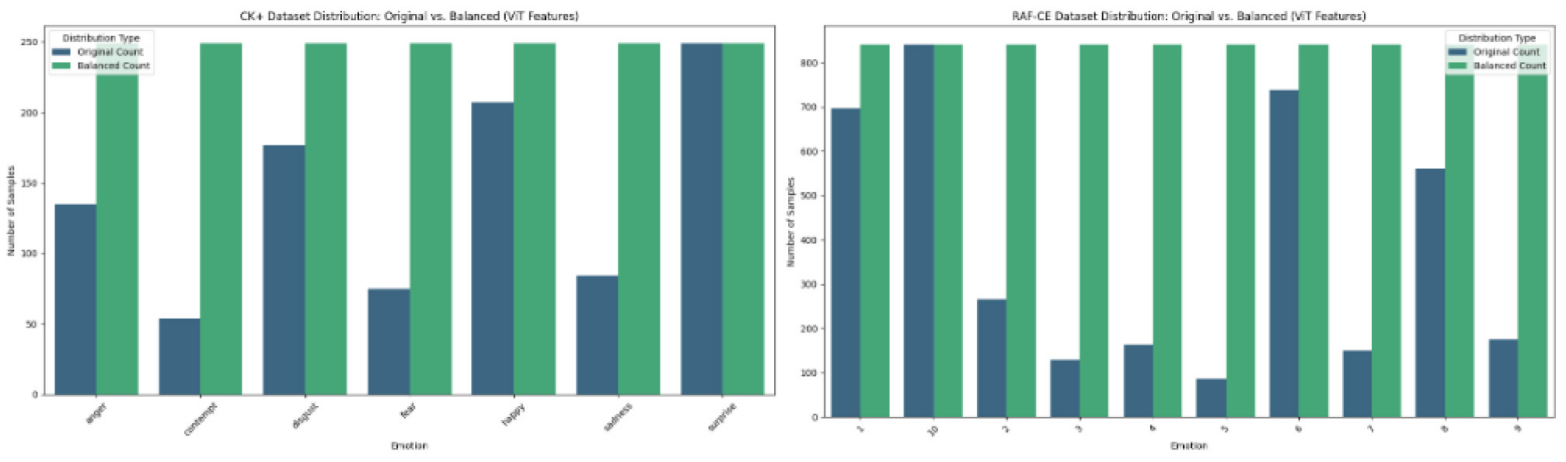
Bar plot of before and after SMOTE samples for CK+ and RAF-CE dataset extracted features.

The bar charts visualize the proactive measures taken to guarantee model performance and fairness. Two extremely unbalanced datasets were successfully converted into resilient, uniform distributions by using SMOTE. High-quality data preparation is shown in Figure 8, where the change from the Original Count (dark blue) to the Balanced Count (bright green) is shown. The original data in both the CK+ and RAF-CE datasets demonstrated a substantial class imbalance; for example, the “5” category in RAF-CE and the “contempt” category in CK+ were underrepresented. The ViT-Base model has an equal chance to learn the distinctive characteristics of each emotion by equalizing these counts. The richness of the training environment has significantly improved in the “After SMOTE” condition. To “fill in the gaps” in the feature space, the minority classes are artificially expanded to match the majority (around 250 samples for CK+ and over 800 for RAF-CE). As seen in your earlier t-SNE graphs, this enables the model to produce more accurate decision boundaries. A dedication to technical excellence is demonstrated by the consistency of the green bars, which ensure that the neural network receives a steady, consistent learning input during the training phase.

### 4.4 Spike generation for CK+ and RAF-CE dataset from the extracted features

#### 4.4.1 Rate encoding

**Figure 9:**
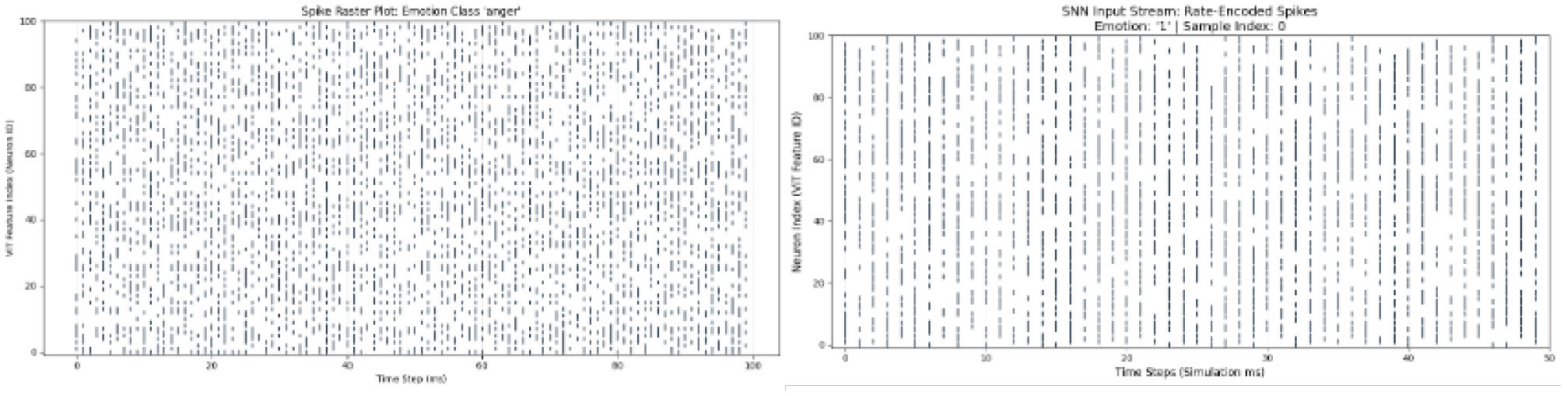
Spike generation using rate encoding for CK+ dataset **(Left image)**, Spike generation using rate encoding for RAF-CE dataset **(Right image)**.

The rate encoding of features from a ViT for SNN processing is depicted in the two spike raster graphs, though their temporal scales and densities differ significantly. Left side of the Figure 9 shows the spike generation for CK+, for the “anger” class, which shows a very dense and homogeneous distribution of spikes throughout the 100 neuron indices during a duration of 100 ms. This indicates a steady firing rate, with the continuous input values over the course of the simulation window high enough to cause numerous spikes across the majority of feature channels. The RAF-CE dataset is shown in right side of Figure 9, compressed into a shorter 50 ms window. Compared to the CK+ picture, the spike distribution appears somewhat more stochastic or varied across individual neurons, while maintaining a similar structural approach that maps feature importance to firing frequency. The transformation of static spatial features into a temporal binary stream, in which the “information” is carried by the density of vertical ticks (spikes) over time, is a basic SNN principle illustrated in both representations. The RAF-CE plot shows a faster, higher-intensity input stream, whereas the CK+ plot provides the network with a larger temporal buffer to integrate these signals.

#### 4.4.2 Threshold encoding

**Figure 10:**
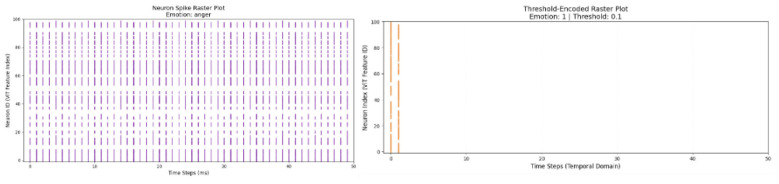
Spike generation using threshold encoding for CK+ dataset **(Left image)**, Spike generation using threshold encoding for RAF-CE dataset **(Right image)**.

These two raster plots, most likely generated from data trained on a ViT, show various results of threshold-based encoding in SNNs. Although they both show how continuous data is transformed into discrete spikes over 50 time steps, their temporal activity and data density levels are essentially different. Figure 10 left side displays a dense, highly repeated firing pattern for the full 50 milliseconds. At nearly every time step, every neuron (indexed 0–100) appears to spike. This implies that, over the course of the temporal window, the input features for the emotion “anger” continuously exceed the encoding threshold. This provides a steady signal but lacks temporal “sparsity,” one of SNNs' main efficiency advantages. A very sparse representation is evident in the second plot Figure 10 right side. Nearly all of the spiking activity is concentrated at the start of the series (time steps 0–1), with no activity for the rest of the sequence. This suggests that either the signal rapidly declined or stabilized below the 0.1 threshold, or the characteristics for “Happily surprised” only activated the threshold mechanism at the first onset.

### 4.5 Hyperparameter tuning

The hyperparameter values for the S-SVM classifier are identified. The generated spikes are taken as input to the S-SVM classifier. The classifier is validated using 5-fold cross-validation and surrogate gradient. Table 1 shows the hyperparameter values tuned for the LIF and QIF-based S-SVM classifier. The dataset was divided into training, validation, and testing sets using a [70-10-20] split ratio to provide a reliable and repeatable assessment of the suggested model. During the training phase, a [k-fold] cross-validation technique [where k = [5]] was used to reduce the danger of overfitting and guarantee generalization over various data distributions. The network was trained using the Adam optimizer with a maximum of [150] epochs, a batch size of [32], and an initial learning rate of (α) = 10^−3^. To find the best configuration for the model's architecture, hyperparameter tuning was methodically carried out using a Grid Search technique. In particular, the tuning procedure maximized both the number of hidden units and the learning rate throughout a range of 10^−3^. In order to provide a strict and empirically supported basis for the final model deployment on the independent testing set, the final hyperparameter values were chosen based on the configuration that produced the maximum accuracy on the validation set.

**Table 1:** Hyperparameter values for LIF and QIF neuron models across datasets.

Dataset	Neuron	Hyperparameter	Value
RAF-CE / CK+	LIF	Timestep	50
		Beta	0.95
		Learning rate	1e-4
		Epoch	100
		Batchsize	32
		Kernel	Rbf
RAF-CE / CK+	QIF	Timestep	50
		Beta	0.95
		Alpha	0.80
		*V*_*rest*_	0.85
		Learning rate	1e-4
		Epoch	100
		Threshold	0.1
		Batchsize	32
		Kernel	Rbf

A dual technique of data-driven initialization and grid-search optimization during K-fold cross-validation is used to determine the hyperparameter configuration for the neuromorphic classifier, namely the leakage factor (β) and the firing threshold (θ) for the LIF and QIF neurons. The leakage factor β, which controls the temporal decay of the membrane potential, is evaluated within the biologically reasonable range of [0.95]. At the same time, the firing threshold θ is structurally scaled in relation to the previous ViT feature extraction layer's normalized output variance. An ideal trade-off is achieved by setting θ to 0.1, which keeps the network highly sensitive to the subtle spatial-temporal muscle transitions typical of compound facial expressions while preventing premature neuron saturation.

### 4.6 Performance metrics

The different performance metrics are used for emotion recognition. Whether emotions are considered discrete categories or continuous dimensions affects performance measures for emotion recognition. The metrics used for this model are listed below:

i. Accuracy: The most fundamental metric in emotion identification is accuracy, which computes the proportion of all right predictions as shown in Equation 18.
$$\begin{array}{l} \text{Accuracy}=\frac{\text{TP}+\text{TN}}{\text{TP}+\text{TN}+\text{FP}+\text{FN}} \end{array}$$ (18)
ii. Recall: Recall, sometimes referred to as the True Positive Rate or Sensitivity, gauges how well a model can identify all pertinent examples of a particular emotion. Recall is calculated using the Equation 19.
$$\begin{array}{l} Recall=\frac{TP}{TP+FN} \end{array}$$ (19)
iii. Precision: The accuracy of the model's positive predictions is gauged by precision, which is sometimes referred to as positive predictive value. which can be calculated by the Equation 20.
$$\begin{array}{l} Precision=\frac{TP}{TP+FP} \end{array}$$ (20)
iv. F1 Score: The most popular statistic for emotion identification is the F1 score as shown in Equation 21. The harmonic mean of precision and recall is known as the F1 Score.
$$\begin{array}{l} F_{1}\text{score}=2\times \frac{\text{Precision}\times \text{Recall}}{\text{Precision}+\text{Recall}} \end{array}$$ (21)

In addition to the above-mentioned performance metrics, energy calculations are performed for this model to demonstrate energy efficiency. As the S-SVM is a hybrid model of a spike neural network and a support vector machine, energy efficiency is calculated to demonstrate that the model can be implemented in hardware. The main factor driving SNNs energy efficiency is their event-driven nature: processing occurs only when a neuron passes a threshold and fires a “spike.” The Sparsity Factor (α) quantifies the network's level of “quietness.” It shows how many neurons fire a spike at a certain time step in relation to the total number of neurons in the network as shown in Equation 22. A lower sparsity factor (greater sparsity) directly corresponds to lower power usage because SNNs are event-driven and only utilize energy when a spike occurs.


$$\begin{array}{l} \text{Sparsity factor}\left(\alpha \right)=1-\left(\frac{\sum_{i=1}^{N}\sum_{t=1}^{T}S\left(t,i\right)}{N\times T}\right) \end{array}$$ (22)

Where: T- Number of simulation time steps

S(t,i)- spikes generated

N- Dimension of the feature vector (neurons in the encoding layer)

N × T- total number of spikes The main “multiplier” for energy savings is the sparsity factor. Only 10% of an SNN is active if its sparsity factor is 0.1. Because SNNs spend much of their time in a low-energy, sparse state and only consume significant power when pertinent data causes a wave of spikes, they are highly preferred for “always-on” edge devices.

## 5 Results

**Figure 11:**
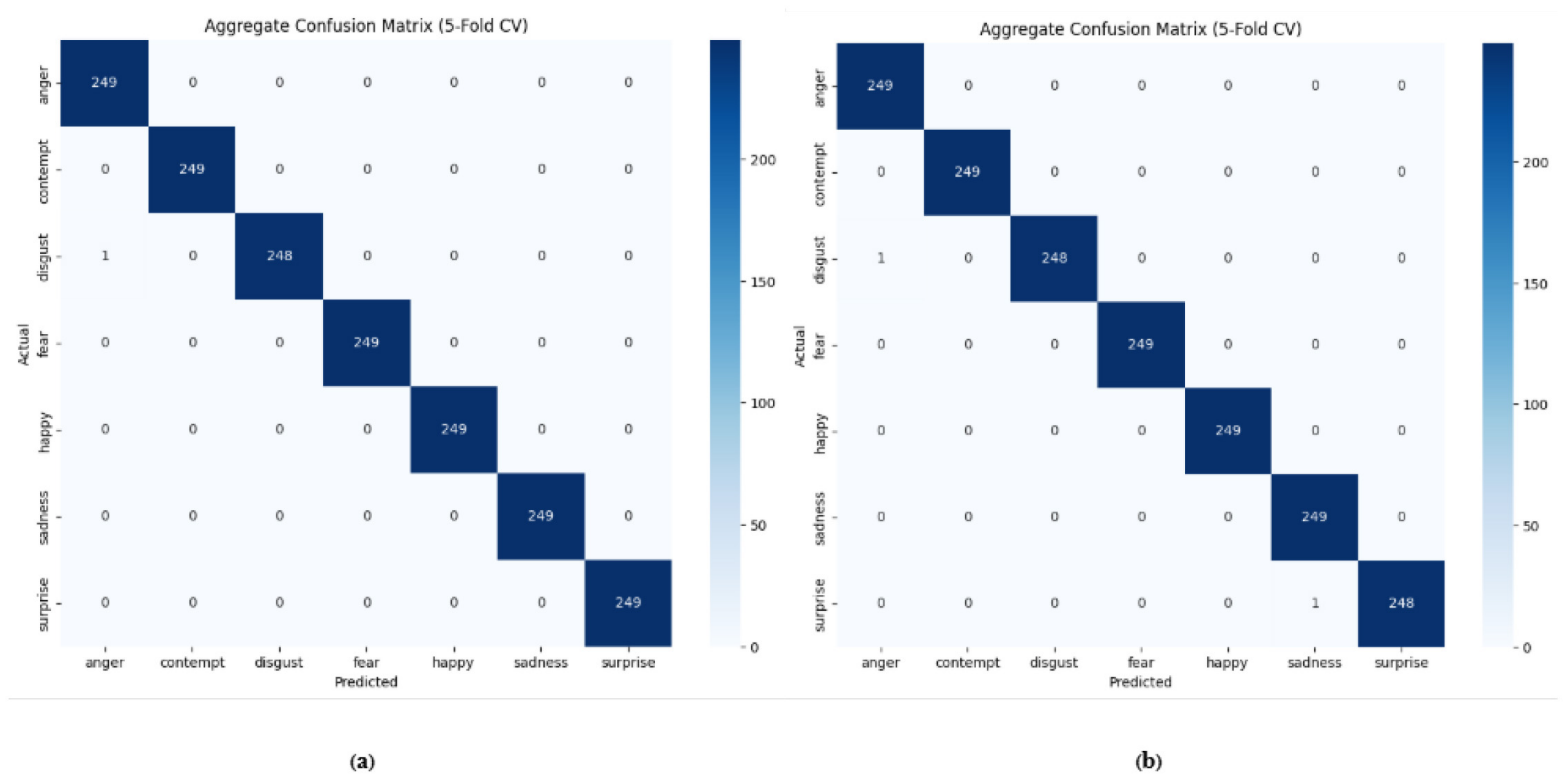
**(a)** Confusion matrix of LIF neuron model with rate encoding for CK+ dataset, **(b)** Confusion matrix of LIF neuron model with threshold encoding for CK+ dataset.

The empirical data collected from several studies shows that neuromorphic spiking models and traditional deep learning architectures have different performance landscapes. Using the LIF neuron model in conjunction with rate encoding, the S-SVM achieves a near-perfect classification accuracy of 99.94% on the controlled CK+ facial expression dataset. There is only one error introduced by this arrangement out of 1,743 total samples, when an occurrence of “disgust” is mistakenly classed as “anger.” Figure 11a shows the confusion matrix for the CK+ dataset using the LIF neuron model and rate encoding for spike generation.

**Figure 12:**
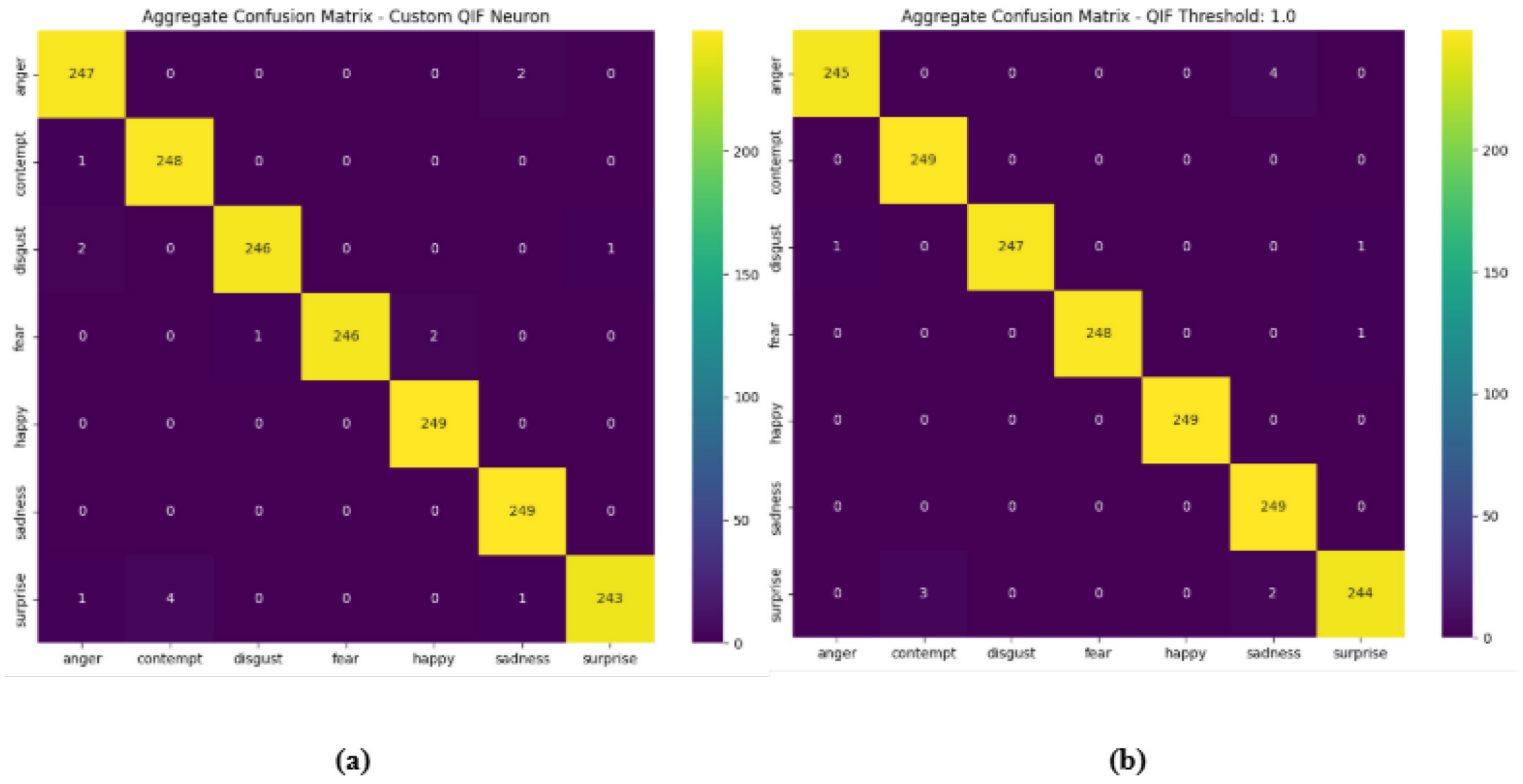
(a) Confusion matrix of QIF neuron model with rate encoding for CK+ dataset, **(b)** Confusion matrix of QIF neuron model with threshold encoding for CK+ dataset.

The accuracy of 99.89% is slightly reduced but still quite dependable when the LIF model is switched to threshold-based spike generation. However, this introduces a second error where a “surprise” sample is incorrectly labeled as “sadness.” Both configurations show a strong concentration of accurate predictions along the major diagonal of their confusion matrices during 5-fold cross-validation, which is reinforced by a deep blue color scaling that shows 248 or 249 accurate classifications out of 249 samples per emotional category. Figure 11b shows the confusion matrix for the CK+ dataset using the LIF neuron model and threshold-based spike generation. On the identical CK+ dataset, the QIF neuron model performs somewhat worse in terms of classification, achieving an accuracy of 99.14% with rate encoding and 99.31% with threshold encoding. Figure 12a shows the confusion matrix for the QIF neuron model with rate encoding. There is some tiny off-diagonal noise in the QIF rate-based matrix, especially when the “surprise” class is incorrectly classified as “contempt,” “anger,” or “sadness,” and there are also small overlaps between “disgust” and “anger.” While the threshold-based QIF model shows better consistency in the “fear” and “contempt” categories, it is more likely to incorrectly identify “anger” as “sadness” in four different cases. “Happy” and “sadness” continue to be the most frequently identified emotional states across both QIF versions, attaining 100% accuracy in numerous test sessions. Figure 12b shows the confusion matrix for the QIF neuron with threshold encoding.

**Figure 13:**
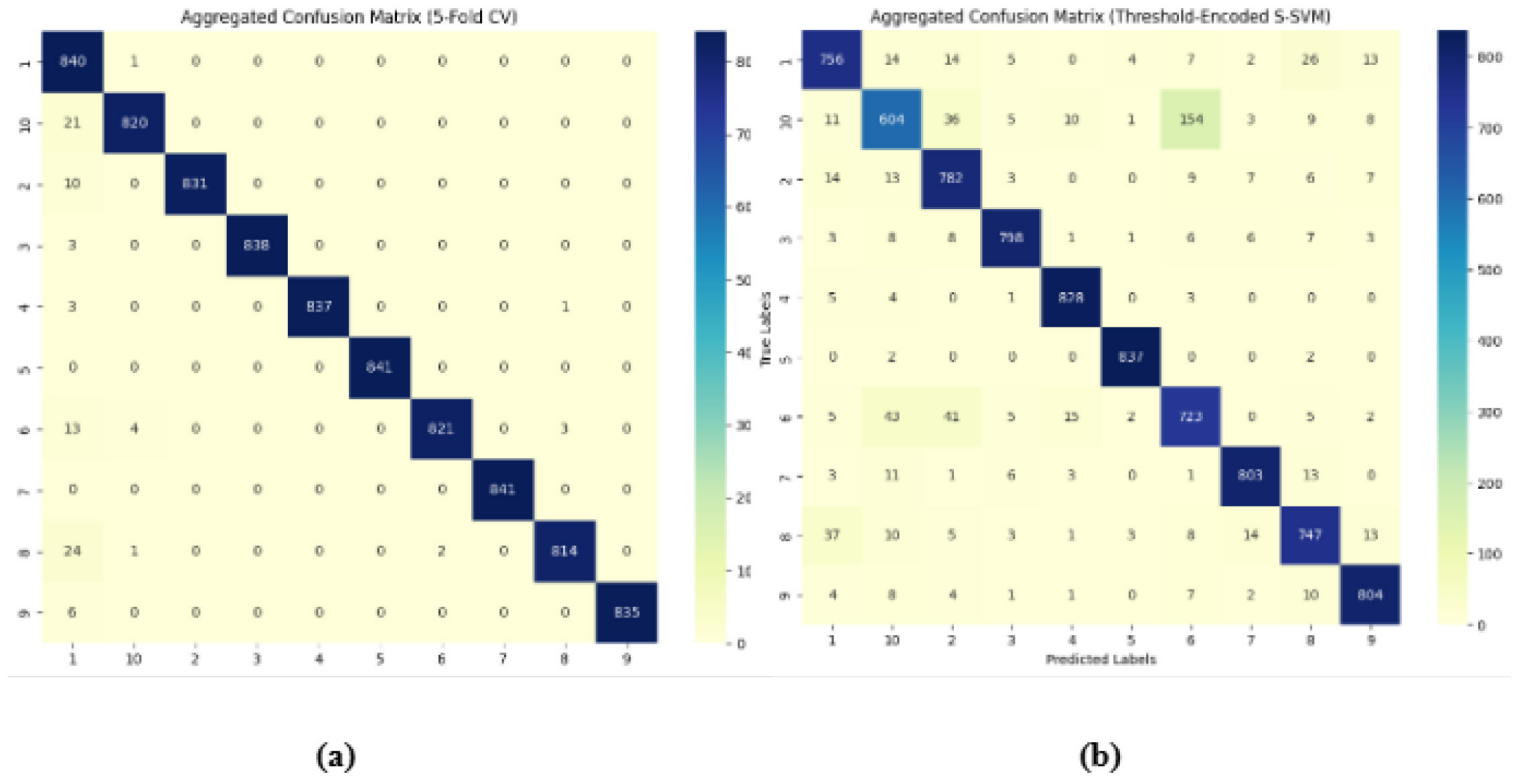
**(a)** Confusion matrix of LIF neuron model with rate encoding for RAF-CE dataset, **(b)** Confusion matrix of LIF neuron model with threshold encoding for RAF-CE dataset.

The performance difference between the architectures and encoding approaches increases dramatically when the models are exposed to the more difficult, unrestricted real-world images of the RAF-CE dataset. By maintaining a high classification accuracy of 98.91% and a precision score of 98.98%, with small errors closely grouped around Class 1, the LIF model utilizing rate encoding exhibits strong stability. Nevertheless, the accuracy of the LIF model deteriorates noticeably when threshold encoding is used, dropping to 91.34% and introducing significant noise across the off-diagonal cells; the confusion matrix is shown in Figure 13a.

**Figure 14:**
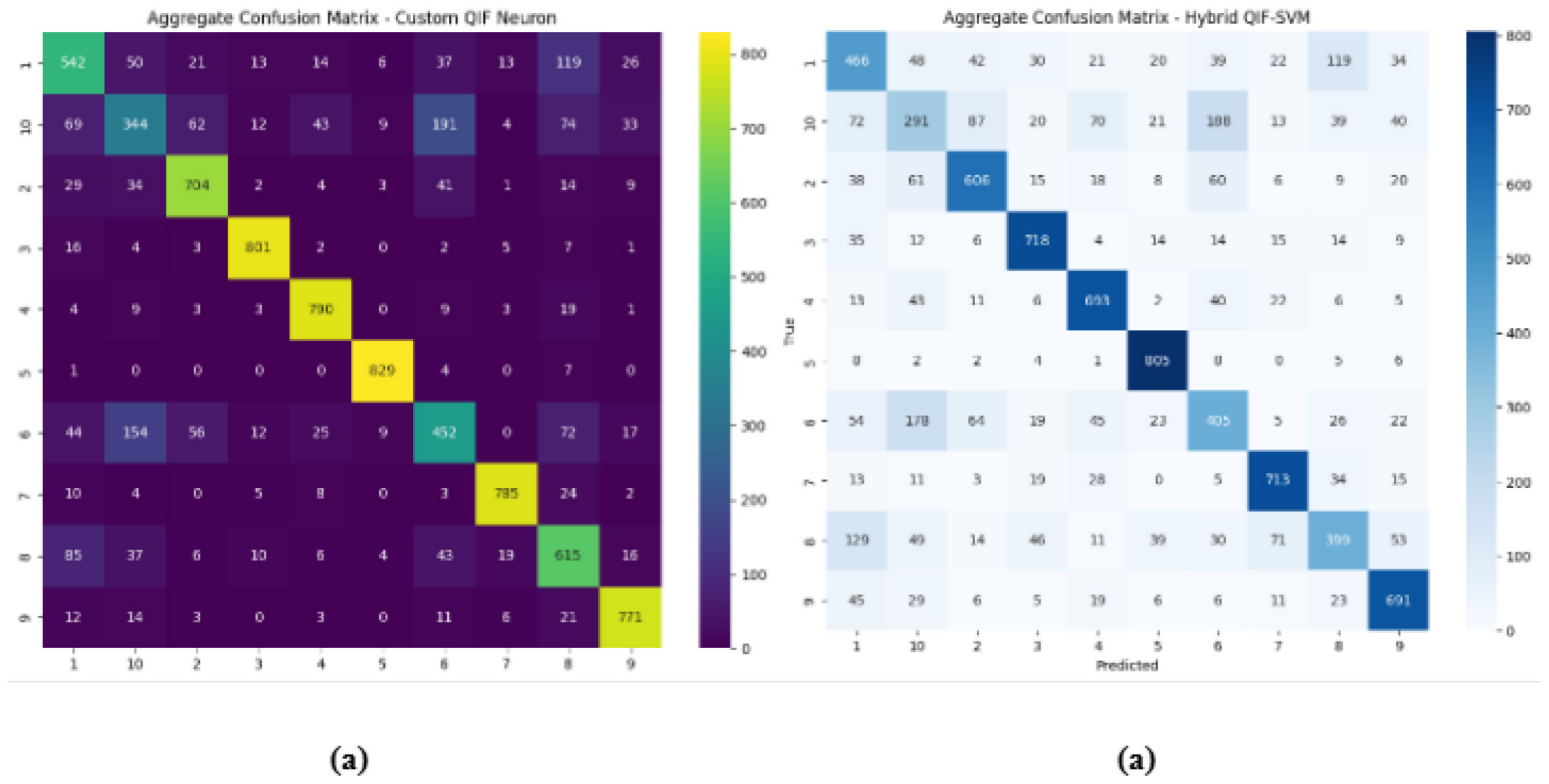
**(a)** Confusion matrix of QIF neuron model with rate encoding for RAFE- CE dataset, **(b)** Confusion matrix of QIF neuron model with threshold encoding for RAF-CE dataset.

On the other hand, the threshold-encoded model in Figure 13b shows far more “noise” and greater variance among the off-diagonal elements. The largest decrease is seen in Class 10, where 154 cases, or almost 20% of the whole class, are mistakenly categorized as Class 6. Errors are also widely distributed over Classes 6, 10, and 2. Table 2 compares LIF and QIF neurons with rate and threshold encoding across the CK+ and RAF-CE datasets. On the RAF-CE dataset, the QIF neuron model's classification stability declines even more sharply, falling to 78.87% accuracy under rate encoding. In comparison, the threshold-based hybrid QIF-SVM framework's accuracy falls to 68.87%. A bottleneck between Class 10 and Class 6, where 191 cases in the rate-coded form and 188 cases in the threshold-coded variant are incorrectly classified, is the result of considerable interclass confusion in both QIF variations. Figure 14a shows the confusion matrix for the QIF neuron with rate encoding. Furthermore, although baseline performance for Classes 5 and 3 is still fairly stable in the 700–800 true-positive range, the threshold-coded QIF model reveals a substantial structural overlap between Class 8 and Class 1, leading to 129 misclassifications and a wide distribution of errors across nearly all off-diagonal cells. Figure 14b shows the confusion matrix for the QIF neuron with rate encoding.

**Table 2:** Comparison table for LIF and QIF neuron models.

Dataset	Encoding techniques	LIF neuron (accuracy)	QIF neuron (accuracy)
CK+	Rate	99.94%	99.14%
	Threshold	99.89%	99.31%
RAF-CE	Rate	98.91%	78.87%
	Threshold	91.34%	68.87%

The network's algorithmic profiling data onto common neuromorphic hardware benchmarks in order to verify the practical viability of the suggested hybrid ViT-S-SVM framework on resource-constrained edge systems is mapped. Specifically, we assessed metrics compatible with specialized event-driven processors like Intel's Loihi or IBM's TrueNorth. CNNs on conventional von Neumann architectures (such as typical GPUs/CPUs) require continuous, synchronous clock cycles to calculate dense floating-point Multiply-Accumulate (MAC) operations, which results in a “always-on” power drain. Neuromorphic hardware, on the other hand, employs non-von Neumann architecture to carry out asynchronous, event-driven processes in which energy is only used when a neuron causes a spike.

Using two different spike encoding techniques, Table 3 assesses the LIF neuron model's performance on two datasets. Regardless of the encoding technique employed, the model achieves nearly flawless results on the CK+ dataset across all metrics Accuracy, Precision, Recall, and F1 Score though Rate encoding performs marginally better than Threshold encoding at 99.94%. This consistency suggests that the LIF model is quite durable for smaller, controlled datasets such as CK+. The RAF-CE dataset, however, shows that the performance differences between the two encoding methods are more pronounced. Threshold encoding shows a discernible deterioration, with metrics falling to the 91% range, whereas Rate encoding maintains strong stability, with accuracy of 98.91% and precision of 98.98%. This decline implies that the LIF model benefits more from the temporal subtleties recorded by Rate encoding than from the constant Threshold approach when dealing with more complex and varied face expressions. Overall, the chart shows that although the LIF neuron is an effective classifier, the choice of spike encoding has a significant impact on its effectiveness on complicated datasets.

**Table 3:** Performance metrics for the CK+ and RAF-CE dataset with LIF neuron model.

Dataset	Encoding technique	Accuracy	Precision	Recall	F1 score
CK+	Rate	99.94%	99.94%	99.94%	99.94%
	Threshold	99.89%	99.89%	99.89%	99.89%
RAF-CE	Rate	98.91%	98.98%	98.91%	98.92%
	Threshold	91.34%	91.36%	91.34%	91.28%

The presented data in Table 4 reveals a substantial paradigm change between neuromorphic computing and conventional deep learning. There is a significant architectural difference between neuromorphic computing and conventional deep learning when the hardware performance metrics are evaluated at a macro level. On the CK+ dataset, the S-SVM with the LIF model achieves a high accuracy of 99.94%, slightly outperforming the traditional Basic CNN with an accuracy of 99.71%. The network Sparsity Factor, however, is what makes a difference. The S-SVM models achieve an amazing network sparsity of 99.52%, which means that the system only fires spikes when important face data is explicitly identified. For the great majority of its processing cycles, the system is completely silent and uses almost little power. On the other hand, the Basic CNN displays a minimal sparsity factor of 0.0202% while operating in a “always on” state. The S-SVM design requires about 5,000 times less operational network activity than the dense, continuous mathematical computations required by the traditional CNN. This operational difference results in a significant reduction in computational overhead.

**Table 4:** Energy-efficient sparsity factor for LIF neuron-based rate encoding method and traditional CNN model.

Dataset	Model	Accuracy	Sparsity factor
CK+	S-SVM (LIF neuron model) + Rate encoding	99.94%	99.52%
	Basic CNN model	99.71%	2.02%
RAF-CE	S-SVM (LIF neuron model) + Rate encoding	98.91%	99.60%

## 6 Discussion

The experimental results demonstrate that spiking architectures can significantly reduce computational overhead without sacrificing the accuracy of facial emotion identification, revealing a significant paradigm shift between neuromorphic computing and traditional deep learning. This operating efficiency is mostly due to the Leaky Integrate-and-Fire (LIF) neuron model's special integration process. Regardless of whether the incoming visual data contains pertinent expressions or static, redundant backdrops, conventional convolutional neural networks are mathematically designed to be constantly active, requiring dense matrix multiplications across every single node and connection. By functioning as an event-driven integrator, the LIF-based S-SVM gets around this inefficiency. Over time, it gathers incoming electrical impulses and only executes calculations when the integrated signal is powerful enough to surpass a predefined threshold. The enormous 99.52% sparsity factor seen in the data is caused by this underlying dynamic, which enables the network to remain completely silent for most of the processing cycle. This sparse processing translates into significant power savings in physical silicon because power consumption in specialized neuromorphic circuitry is directly proportional to the total number of spikes generated.

A crucial theoretical contrast in neuron dynamics is further highlighted by the combined results, which show that the LIF model's linear-leaky characteristics are intrinsically better than the QIF model's non-linear dynamics for challenging classification tasks. The RAF-CE dataset's unconstrained variance makes the non-linear acceleration of the QIF membrane potential extremely sensitive to small background variations, whereas both models readily map the clear, localized data of the CK+ dataset. This vulnerability causes severe interclass bottlenecks, especially between Class 10 and Class 6, by obfuscating the feature borders between close emotional classes. In contrast, stochastic background noise is automatically dampened by the LIF model's linear leak, enabling the succeeding SVM layer to create clear, very different separating hyperplanes. Additionally, the analysis shows that spike encoding methods cannot be evaluated without reference to the neuron model. The decline in LIF performance from 98.91% under rate encoding to 91.34% under threshold encoding indicates that the continuous intensity gradients maintained by rate frequency maps are crucial for complicated real-world facial datasets. The hybrid QIF-SVM framework produces widespread off-diagonal error noise when it is binarized too quickly via thresholding, making it difficult to distinguish overlapping facial features. The hybrid S-SVM model resolves a number of SNNs' historical shortcomings when compared to earlier research and more general neuromorphic milestones. When converting conventional networks or employing spiking backpropagation, traditional deep SNN implementations—such as those based on deep multi-layered Spiking-VGG or Spiking ResNet topologies—often experience significant accuracy loss, frequently falling to the 75% to 82% accuracy range on complex datasets like RAF-DB due to error accumulation across deep layers. This hybrid model completely avoids the problems of exploding and vanishing gradients by combining the maximum-margin classification power of a Support Vector Machine with the sparse feature-extraction capability of a LIF processing step. This allows the model to maintain a highly resilient 98.91% accuracy under real-world conditions. The discipline of Edge-AI will greatly benefit from its capacity to maintain a high degree of accuracy while reducing computational activity by an astonishing 5,000 times. It provides a verified hardware architecture blueprint for implementing high-precision, real-time emotion recognition systems on battery-powered devices, such as wearable assistive technology, automotive cabin monitoring frameworks, and localized robotics, where the use of conventional, power-hungry deep learning networks is totally impractical due to strict thermal envelopes and rigid power constraints.

Beyond localized hardware limitations, the LIF-based S-SVM's unparalleled efficiency and micro-second responsiveness create revolutionary opportunities for further study and application in a variety of technical and socioeconomic fields, most notably affective computing and human-computer interaction (HCI). Real-time affective computing has traditionally been limited to cloud-tethered setups or high-power workstations by traditional deep learning implementations of FER. This work provides a workable foundation for incorporating continuous, compassionate AI into low-power consumer products by showcasing an architecture that can sustain strong performance within sub-milliwatt power limitations. This low-overhead architecture may support procedural story production and dynamic difficulty modification in gaming and virtual environments by using localized edge-processing to non-invasively track player irritation, immersion, or joy. For widespread use in user-centric HCI systems, our deployment paradigm guarantees total localized data privacy a crucial ethical milestone. Real-world facial expressions are rarely consistent; instead, they are greatly influenced by behavioral variances, display standards, and cultural settings. As a result, our model serves as a scalable, energy-efficient engine for worldwide, culturally sensitive human-agent collaboration and offers a very robust computational basis for future research into how localized spiking patterns adapt to multi-ethnic micro-expressions (Gursesli et al., 2026).

## 7 Conclusion

The proposed study shows that a S-SVM framework offers a highly efficient and biologically inspired solution for FER by combining an S-SVM classifier with a ViT for feature extraction. The primary architectural innovation, which combines a high-capacity ViT feature extractor with a specially designed S-SVM classifier, solves a significant problem in the literature: the precise parsing of intricate facial expressions without the significant computational overhead associated with traditional deep networks. Through a comprehensive evaluation utilizing K-fold cross-validation, the study confirms that the LIF neuron model consistently outperforms the QIF model in terms of accuracy and resilience. The LIF neuron maintains an amazing accuracy of 98.91% on the complex, real-world RAF-CE dataset, while the QIF neuron's performance sharply declines. On the controlled CK+ dataset, both neurons do exceptionally well, though. When contrasting the Sparsity Factor of these neuromorphic models with traditional architectures, the biggest difference becomes apparent. Beyond classification precision, the S-SVM architecture offers unparalleled energy efficiency with sparsity factors of 99.52% for CK+ and 99.60% for RAF-CE. A Basic CNN model, on the other hand, obtains a sparsity factor of just 0.0202%, which means that it is practically “always on” and executing complex computations throughout its whole network for each input. In contrast to the event-driven S-SVM, which is “silent” and inactive for more than 99% of the time, the CNN's lack of sparsity causes a significant computational cost even though it retains a high accuracy of 99.71%.

These results show that compared to traditional deep learning models that process data redundantly, the LIF-based SNN's event-driven architecture can filter complicated emotional data with substantially less “brain activity,” using significantly less power. Ultimately, this work shows that the best architecture for developing high-accuracy, low-energy FER systems that can be implemented in real-time, resource-constrained settings where a traditional CNN would be too power-hungry to continue operating is the LIF neuron in combination with an S-SVM. This work offers a useful, real-time blueprint for deploying reliable emotion-recognition systems directly onto resource-constrained neuromorphic hardware where conventional CNNs would instantly fail by demonstrating that the combination of a ViT, LIF neuron dynamics, and an S-SVM classifier can match deep learning precision while operating on a fraction of the power budget.

## Data Availability

Publicly available datasets were analyzed in this study. This data can be found here: https://www.kaggle.com/datasets/davilsena/ckdataset and http://whdeng.cn/RAF/model4.html.
